# Environmental Heterogeneity Imposed by Photovoltaic Array Alters Grassland Soil Microbial Communities

**DOI:** 10.1111/gcb.70376

**Published:** 2025-07-23

**Authors:** J. Alexander Siggers, Matthew A. Sturchio, Lillian Gordon, Shelby Mead, Melinda D. Smith, Alan K. Knapp

**Affiliations:** ^1^ Department of Biology and Graduate Degree Program in Ecology Colorado State University Fort Collins Colorado USA; ^2^ Department of Natural Resources and the Environment Cornell University Ithaca New York USA; ^3^ Department of Wildland Resources and the Ecology Center Utah State University Logan Utah USA; ^4^ Department of Ecosystem Science and Sustainability Colorado State University Fort Collins Colorado USA

**Keywords:** belowground functionality, grassland, microbial biomass, photovoltaics, soil microbiome, spatial heterogeneity

## Abstract

The rapid expansion of photovoltaic (PV) energy production has generated concern over its potential ecosystem impacts. PV arrays induce unique microenvironmental conditions by altering resource availability and substantially impacting aboveground processes. However, the belowground consequences of PV development are understudied, limiting our understanding of overall ecosystem impacts. Here, we paired soil physiochemical, molecular, and functional analyses with aboveground measures to assess plant–soil–microbial responses to distinct microsites beneath a single‐axis tracking PV system in a semi‐arid C_3_ grassland. We hypothesized that each PV microsite would harbor a unique suite of soil physiochemical properties and microbiomes. We found only subtle differences in soil organic matter and pH, corresponding with aboveground productivity patterns, but other physiochemical properties remained unchanged. However, soil microbial community structure and function differed markedly across PV microsites and from a reference grassland plot. Within the array, microbial decomposition rates were highest where plant productivity and organic matter were greatest, but surprisingly lowest where soil moisture remained elevated throughout the growing season. Overall, these findings suggest that PV arrays create disparate patterns of soil microbial community structure and function, which may feedback to influence overall ecosystem functionality. Coarse measures of soil physiochemical properties, such as total carbon, may overlook key impacts of PV development.

## Graphical abstract

Photovoltaic (PV) energy generation is a key solution to reducing greenhouse gas emissions, however there are major concerns regarding the ecosystem impacts of PV development. Leveraging a ground‐mounted PV array in a Colorado grassland, we found that soil biotic properties, such as soil microbial community composition, function, and biomass, differ between PV microsites, while soil physiochemical parameters showed negligible differences. We highlight the importance of limiting soil disturbance during PV development and the need for further studies across unique ecosystems.

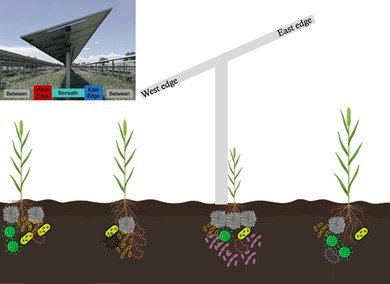

## 1 Introduction

Transitioning to renewable and sustainable forms of energy generation is a critical step in avoiding the most extreme consequences of anthropogenic climate change (Olabi and Abdelkareem 2022). Solar photovoltaic (PV) arrays are considered to be the best immediate solution for reducing CO_2_ emissions by 2030 (Lee et al. 2023), yet there is often little incentive for solar developers to consider how PV facilities affect ecosystem services provided by underlying soils, plants, and animals (Armstrong et al. 2014; Moore‐O'Leary et al. 2017; Grodsky 2021; Sturchio and Knapp 2023). Given the large spatial footprint of solar energy production, particularly in agriculturally important landscapes (i.e., croplands and grasslands; Adeh et al. 2019), where sustainable land use can be a challenge, legitimate concerns about long‐term land stewardship under solar arrays must be addressed (Hernandez et al. 2014, 2019; Adeh et al. 2019; U.S. Department of Energy [DoE] 2021).

Agrivoltaics is an approach to solar energy generation that accommodates agricultural activities (e.g., row and speciality crops, grazing) between and beneath PV panels (Goetzberger and Zastrow 1982; Dupraz et al. 2011; Barron‐Gafford et al. 2019). This is particularly appealing in grazed perennial grasslands, as vegetation is short‐statured, irradiance is high, and there is less need to operate large machinery in close proximity to PV panels (Pascaris et al. 2022). Although the abiotic controls of grassland ecosystems have been studied in many contexts (e.g., drought, grazing, nitrogen deposition; Knapp et al. 2020; Irisarri et al. 2015; Wei et al. 2013), the environmental heterogeneity imparted by PV panels at small spatial scales represents novel conditions in landscapes typically defined by their relative homogeneity (Knapp and Sturchio 2024). For example, precipitation inputs in PV arrays are either deflected when rain is blocked from reaching the ground directly beneath panels or concentrated when rain is shed to panel edges. In single‐axis tracking arrays where panels track the sun across the sky (east–west), PV‐induced environmental heterogeneity in both light availability to plants and alterations in soil moisture have been shown to substantially alter patterns of productivity, physiology, and phenology across climatologically disparate grasslands (Adeh et al. 2018; Andrew et al. 2021; Vervloesem et al. 2022; Kannenberg et al. 2023; Sturchio, Kannenberg, and Knapp 2024; Sturchio, Kannenberg, Pinkowitz, and Knapp 2024; Knapp and Sturchio 2024).

Generally, heterogeneity in light, temperature, and water availability strongly influences primary productivity (Knapp and Seastedt 1986; Möhl et al. 2020; Sala et al. 1988), which is often tightly linked to belowground communities and the processes they control (Wardle et al. 2004; Bardgett and Wardle 2010). For instance, elevated soil temperature can modify plant growth and rhizodeposition (Kuzyakov et al. 2007), while subtle shifts in soil moisture can favor particular functional groups of soil bacteria and fungi that may in turn influence plant nutrient availability (Kaisermann et al. 2015; Borowik and Wyszkowska 2016; Fierer 2017). Even without plants, abiotic alterations can be a primary determinant of belowground processes (Jackson et al. 2017). Although it is well known that PV arrays create uniquely dynamic environments, we currently lack a fundamental understanding of how altered abiotic drivers influence aboveground–belowground linkages and overall belowground processes within solar facilities.

Previous belowground investigations have focused on how PV arrays induce variation in soil moisture, soil temperature, and several other edaphic factors (Adeh et al. 2018; Barron‐Gafford et al. 2019; Choi et al. 2020, 2023; Andrew et al. 2021; Carvalho et al. 2025). Soil nutrient availability and total carbon (C) have been shown to be consistently lower within PV arrays relative to nearby control sites, while other soil properties, like pH and organic matter (OM) shift unpredictably (Choi et al. 2020, 2023; Lambert et al. 2021; Moscatelli et al. 2022). Such responses may be ecosystem dependent and driven by varying construction practices used to establish the arrays. Evaluations of in situ belowground biotic function, including soil respiration, soil microbial community structure, and overall microbial activity via extracellular enzyme assays, have also been performed in solar arrays, but only at limited microsites (between and beneath panels, Lambert et al. 2021; Moscatelli et al. 2022; Li et al. 2023; Carvalho et al. 2025; Scholten et al. 2025), thus overlooking the wider range of soil microenvironments within PV facilities (see Choi et al. 2020; Sturchio et al. 2022; Sturchio, Kannenberg, Pinkowitz, and Knapp 2024). Hence, there is a major gap in understanding the full spectrum of consequences for soil abiotic heterogeneity within PV arrays, contributing to a more general lack of understanding of how this projected land use change will alter belowground biological activity.

To better understand how PV‐induced environmental heterogeneity alters key soil properties and processes that underpin biogeochemical cycling and ecosystem function, we paired soil functional assays with physiochemical and molecular analyses in a semi‐arid, C_3_ dominated grassland. The array selected for our study was particularly valuable because a single graminoid species dominated all microsites, allowing us to isolate how abiotic environmental heterogeneity impacted soil processes in the absence of plant community compositional shifts. We assessed decomposition rates of organic compounds, quantified microbial biomass carbon (C) and nitrogen (N) and microbial community structure, and performed a suite of physiochemical analyses (e.g., pH, total C, electrical conductivity) to determine relationships between PV microsites and soil properties and processes. We hypothesized that PV‐induced differences in soil physiochemical profiles would drive contrasting soil microbial community structure and function across PV microsites, with the most substantial differences between microsites where the greatest previously documented differences in abiotic variation occur (e.g., west panel edge and beneath panels, Choi et al. 2020; Sturchio et al. 2022; Sturchio and Knapp 2023) relative to undisturbed grassland. Specifically, we expected that soil microbial biomass and decomposition rates would be highest along the west panel edge, where soil moisture is greatest throughout the growing season, and lowest beneath panels due to the lack of light, productivity, and precipitation inputs. We further expected that soil microbial community structure beneath panels would differ markedly from other PV microsites and control plots.

## 2 Materials and Methods

### 2.1 Study Site

**FIGURE 1:**
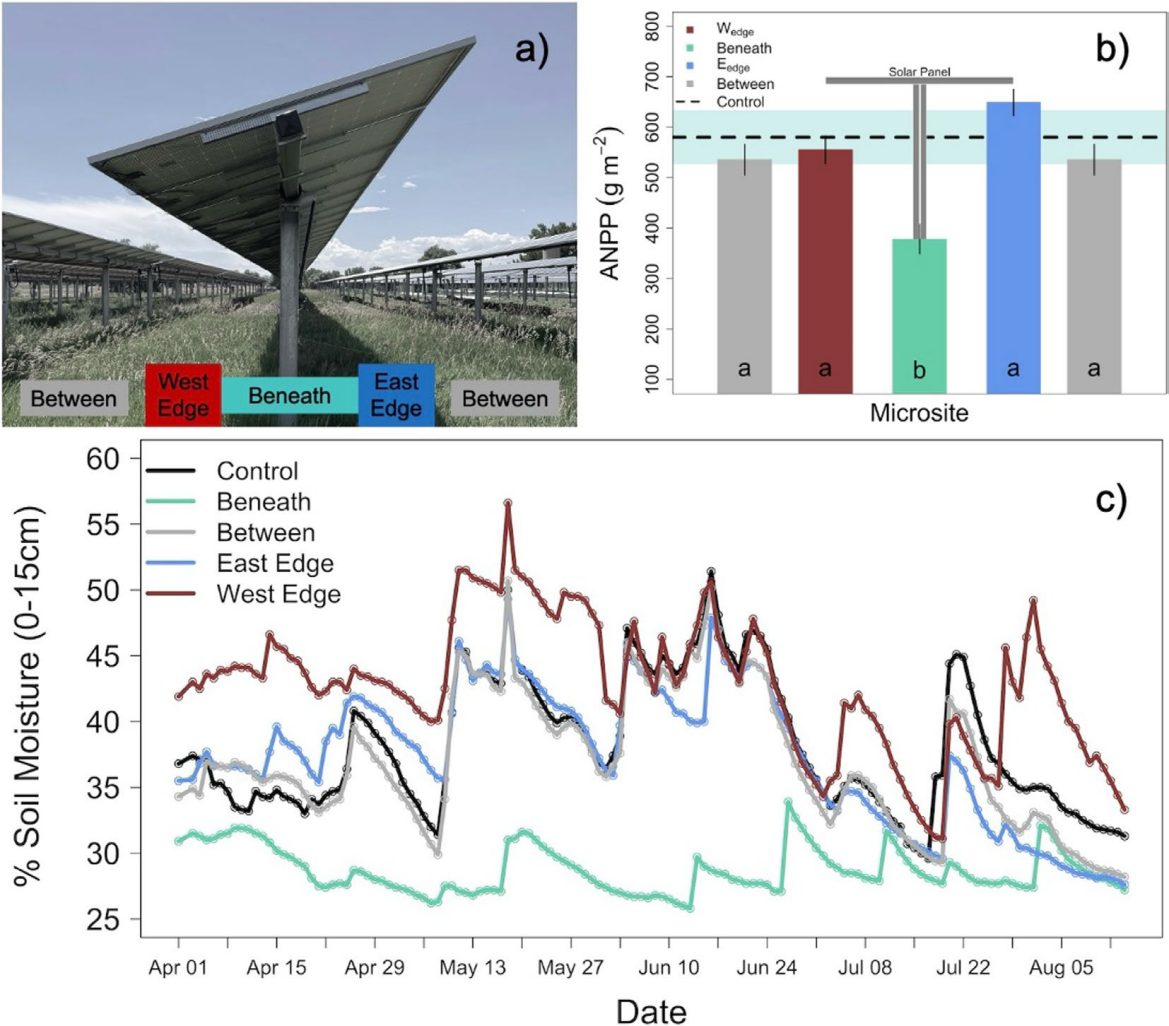
Distribution of experimental microsites, along with their impacts on ANPP and soil moisture. (a) Experimental microsites (between, west edge [W_edge_], beneath, east edge [E_edge_]) for sample collection in the perennial C_3_ grassland at Jack's Solar Garden. Between microsites are shown twice to display spatial orientation of microsites, which is also included in subsequent figures. (b) Microsite patterns of mean ANPP for 2023 are color coded to match (a). Lines on vertical bars represent standard error from the mean. Letters in bars represent statistical differences (*p* < 0.05, Table S1). The control site mean ANPP is represented by a dashed horizontal line with a light blue band representing standard error from the mean, which is repeated in the figures below. (c) Average growing season volumetric soil moisture (0–15 cm).

Research was conducted at Jack's Solar Garden (JSG) in Longmont, Colorado, USA (40.12191, −105.12936). JSG was established in 2019 as a 1.2 MW solar energy production and research facility fabricated with single‐axis tracking panels (tracking east–west). Panels are 2 m long (east–west) and mounted 1.8 m above the ground when flat, with 3.2 m of interspace between the western edge of a panel row and the eastern edge of the next (Figure 1). The land was not graded during array installation and overall disturbance was minimized such that grass cover is 100% throughout the array. The soils at the site are Nunn sandy clay loam (of the Nunn series) with 0%–1% slopes (Soil Survey Staff, Natural Resources Conservation Service, United States Department of Agriculture 2025). The soils are well‐drained and taxonomically classified as fine, smectitic, mesic Aridic Argiustolls formed primarily from alluvial and aeolian deposition with a predominantly loamy alluvium parent material (National Cooperative Soil Survey 2025). The vegetation beneath panels is primarily dominated (> 90% cover) by a monoculture of *Bromus inermis* , a naturalized perennial C_3_ grass. Other species, such as *Medicago sativa* (Alfalfa), *Dactylis glomerata* (C_3_ Orchard grass), and *Tragopogon dubius* (C_3_ forb), compose the majority of subordinate plant species, although they were not present in any of the experimental microsite plots. JSG was formerly managed as a hay farm until PV installation in 2019, when active management (e.g., mowing and fertilization) ended. The site is situated at 1508 m of elevation, with a semi‐arid climate, a mean annual temperature of 9.7°C, and 365 mm of annual precipitation (Colorado Climate Center; http://ccc.atmos.colostate.edu/).

### 2.2 Experimental Design

In May 2023, we established four 15.6 m transects perpendicular to the rows of PV panels within a portion of JSG that is composed of undisturbed perennial grassland (Figure S1). Each transect was split into three replicate sampling areas comprised of four unique PV microsites based on past studies in single‐axis tracking arrays that have identified these as areas with unique light and precipitation inputs (Kannenberg et al. 2023; Sturchio, Kannenberg, and Knapp 2024; Sturchio, Kannenberg, Pinkowitz, and Knapp 2024; Sturchio and Knapp 2025; Figure 1a). This resulted in 48 experimental microsite plots (1 m × 0.5 m), with 12 per microsite treatment (Figure S1, Sturchio and Knapp 2025). The between panel (*Between*, directly between PV support posts) microsites receive precipitation inputs similar to control plots but receive only ~70% of direct sunlight. Rainfall events at panel edges are concentrated due to runoff, but the spatial pattern of precipitation redistribution is time dependent. For example, morning rainfall is shed to the eastern panel edge (E_edge_, 80 cm east of PV support posts) and afternoon rainfall is shed to the western panel edge (W_edge_, 80 cm west of PV support posts). Total sunlight availability is also limited at panel edges, with E_edge_ and W_edge_ receiving ~47% and ~42% of total daily light inputs, respectively. Beneath panel microsites (*Beneath*, directly in line with PV support posts) receive the least rainfall and ~30% of total daily light inputs. Our external control (Control) plots (*n* = 4) were located in an undisturbed area 20 m outside of the solar array and ~100 m from the nearest experimental microsite plot in an area of homologous soil type, slope, and management.

### 2.3 Environmental Measurements

Volumetric water content (VWC, denoted as soil moisture [SM]) was measured using CS616 Soil Moisture Sensors (Campbell Scientific, Logan, UT) at 15 min increments from May to September 2023, hereafter considered the growing season. Each experimental microsite and control plot had paired sensors at 0–15 cm depth (Figure 1). Differences in photosynthetic photon flux density, air temperature, and relative humidity across microsites were previously characterized (Kannenberg et al. 2023; Sturchio, Kannenberg, Pinkowitz, and Knapp 2024), allowing us to infer these values. The mean air temperature on the sampling date was 22.7°C with light overcast conditions.

### 2.4 Soil and Plant Sampling

Destructive soil sampling only took place once on August 2, 2023, near peak primary production, to minimize disturbance and interference with other aspects of the study (i.e., biomass harvest). Three soil samples were collected from each experimental microsite (*n* = 48) and control (*n* = 4) plot (15 cm depth × 2.22 cm diameter) using a steel soil probe (AMS Inc., ID, USA) and homogenized in the field to capture within‐plot spatial heterogeneity. Immediately prior to bulk homogenization, samples for microbial analyses were collected from the interior of the core (5–10 cm depth) using a sterilized metal spatula and homogenized, and each tool was cleaned thoroughly with 70% ethanol after each plot. Samples were collected, placed in a cooler, and immediately transported back to the Colorado State University (CSU) laboratory. The samples collected for microbial analysis were then immediately subset and transferred to −80°C storage for DNA preservation (~3 g per sample) and remaining portions were refrigerated. All analyses requiring fresh soils (e.g., microbial biomass, MSIR) were conducted within a week of sample collection, and DNA was extracted from frozen samples within 2 weeks of collection. All subsequent soil analyses were conducted on a randomly selected subset of samples from experimental microsite and control plots. Soil cores (10 cm depth × 6 cm diameter) were collected in replicate (*n* = 3 per microsite treatment and control) on August 3, 2023, for bulk density measurements.

At the end of the growing season (late‐September), all plots were sampled for aboveground net primary productivity (ANPP). We placed a 0.1‐m^2^ quadrat in each plot and harvested all biomass to ground level. Harvested biomass was dried at 60°C for 72 h, then weighed to the nearest 0.01 g. For experimental microsite plots (*n* = 12 per microsite treatment), variability in sample size was due to interference by native fauna or logistical constraints. For Control plots (*n* = 4), sample size was limited by available space.

### 2.5 Soil Physiochemical Analysis

Soil properties were assessed using conventional methodologies at the Colorado State University SPUR Soil, Water and Plant Testing Laboratory. Gravimetric soil moisture was measured by weighing 10 g of fresh, sieved (2 mm) soil to the nearest 0.01 g, drying at 105°C for 48 h, then reweighing. Soil pH and electrical conductivity were measured in a 1:1 (w:v) soil and water suspension using a pH and conductivity meter. Soil organic matter (SOM) was determined via loss on ignition (Robertson et al. 1999). Soil total C and N were determined via dry combustion on an elemental analyzer using dry soil samples (VELP CN 802, Italy). To prepare for soil total C and N analyses, soils were sieved (2 mm) to remove roots, any remaining roots were extracted with tweezers, then soils were ground with a sterile mortar and pestle. Soil texture was determined via hydrometer and wet sieving methods to calculate the clay and sand fractions, respectively. Soil bulk density was calculated following the drying and weighing of soil cores (10 cm × 6 cm), then dividing dry weight by core volume.

### 2.6 Microbial Biomass

Microbial biomass C and N were assessed via the chloroform fumigation extraction method (Brookes et al. 1985). Biological replicates were randomly selected to represent each experimental microsite treatment and controls (*n* = 8 for E_edge_, W_edge_, and Beneath, *n* = 4 for Between, *n* = 3 for Control), and 10 g of fresh fumigated and unfumigated samples were extracted in 50 mL of 0.5 M K_2_SO_4_ after 30 min of shaking. Dissolved organic C and total N were determined using a Schimadzu TOC‐L (Schimadzu Corp, Japan). Microbial biomass C and N were calculated as the difference between fumigated and unfumigated samples with the coefficients *k*_EN_ of 0.54 and *k*_EC_ of 0.45. We then converted all measures to a per gram dry soil basis using our gravimetric soil water content calculations.

### 2.7 Microbial Substrate‐Induced Respiration

Microbial Substrate‐Induced Respiration (MSIR) rates were generated for randomly subsampled soils of each experimental microsite treatment and control (*n* = 3 biological replicates in duplicate [*n* = 6 per treatment]) using the MicroResp system (Campbell et al. 2003; Creamer et al. 2016). Colorimetric gel detector plates were generated using a cresol red indicator solution to be read on an Infinite 200 Pro plate reader (Tecan, Switzerland) at 570 nm. Five substrates (plus water) were selected based on their common occurrence as native labile and recalcitrant (i.e., length of the C chain) organic carbon inputs. These were: d‐(+)‐glucose, α‐cellulose, d‐(+)‐xylose, *N*‐acetyl‐d‐glucosamine, and lignin (alkali). Water was a sixth substrate to provide basal respiration measurements. Soils (0.3 ± 0.03 g) were loaded into 96‐well plates and stored at 4°C in the dark for 3 days to allow acclimation following disturbance. The five substrates were then added to the soils in six replicate 25 μL aliquots. The plate was left open for a period of 15 min to release any potential carbonates present that may obscure readings following the addition of acidic substrates (Creamer et al. 2016). Baseline colorimetry readings were collected from the indicator plates at 570 nm prior to sealing. Indicator plates were then sealed onto deep‐well plates and incubated at 20°C for 6 h. Colorimetry values were then collected from the indicator plates to calculate respiration rates (μgCO_2_‐Cg^−1^ h^−1^) as the difference between final and initial values. Overall substrate‐induced respiration was calculated by subtracting basal respiration then summing the responses for each sample and substrate (Colombo et al. 2016).

### 2.8 Nutrient Probes

Plant root simulator (PRS) probes (Western Ag Innovations, Saskatoon, Saskatchewan, CA) were used to assess plant available macronutrients (N, P, and K) and micronutrients (Ca, Mg, Fe, Mn, Cu, Zn, B, S, Pb, Al, and Cd). PRS probes imitate plant uptake of ions via paired 10 cm^2^ cation and anion exchange resin membranes inserted in the upper soil profile. We buried probes (*n* = 2–3 pairs) in each plot at two points during the growing season. The first period (May 31–June 26) was chosen to represent the early growing season. The second period (August 2–September 5) was chosen to represent the late growing season. Each pair of probes was retrieved and sent to WesternAg Innovations for processing. The two to three pairs from each plot were eluted together within the laboratory.

### 2.9 Sequencing

Soil microbial DNA was extracted using the Qiagen DNeasy PowerSoil Pro Kit (Qiagen, Hilden, Germany) according to the manufacturer's specifications. All DNA extractions were done at Colorado State University. DNA samples were assessed for quality and quantity using a NanoDrop Lite Spectrophotometer (ThermoFisher Scientific, MA, USA) and Qubit 4 Fluorometer (Invitrogen). Extractions were reiterated if the 260/280 absorbance ratio was below 1.7 due to low DNA quality. Once all DNA extractions were successfully completed and quality checked, they were stored at −20°C until the samples were sent to the CIRES Fierer's Microbial Community Sequencing Lab at the University of Colorado Boulder (Boulder, Colorado) for library preparation and sequencing. DNA was amplified using a SimpliAmp Thermocycler (ThermoFisher Scientific). Illumina MiSeq (Illumina Inc., CA, USA) 2 × 150 bp chemistry at 500 cycles was used for paired‐end amplicon sequencing of the 16S V4 (515F/806R primers; Apprill et al. 2015; Parada et al. 2015) and 2 × 250 bp chemistry at 500 cycles was used for paired‐end amplicon sequencing of the ITS (ITS1/ITS2; Caporaso et al. 2012) regions, with no‐template and extraction controls included in each run.

### 2.10 Bioinformatic Processing

Raw data were received in FASTQ format and demultiplexed using QIIME2 2023.5 software (Bolyen et al. 2019). Sequences were assessed for quality following demultiplexing, merged, then denoised using the DADA2 plug‐in (Bokulich et al. 2013; Callahan et al. 2016). Low‐quality sequences (*Q* < 25) were filtered out. Taxonomy was assigned for all sequences using the feature‐classifier QIIME2 plug‐in (Bokulich et al. 2018). We assigned taxonomy to 16S sequences against the GreenGenes2 database (McDonald et al. 2023) and ITS sequences against the UNITE database (Nilsson et al. 2019) at 99% similarity, generating operational taxonomic units (OTUs). Mitochondrial and chloroplast reads were removed using the taxa‐filter table plug‐in (Bolyen et al. 2019). Feature table and taxonomic assignments were exported for downstream analysis in R software (R Core Team 2017).

### 2.11 Statistical Analysis

Samples were separately analyzed for bacterial/archaeal (16S) and fungal (ITS) communities. OTUs under 20 total reads were removed from analyses to minimize contaminant and sequencing errors (Cao et al. 2021). Samples were rarefied at 11,000 reads for 16S rRNA and 26,000 reads for ITS rRNA. These rarefaction values were chosen to retain the maximum number of samples and allow comparison of samples with differing sequencing depths. Rarefied OTU tables were used for alpha diversity calculations, but not for other downstream analyses. The ‘mctoolsr,’ ‘vegan,’ and ‘phyloseq’ R packages were used to analyze microbial communities (McMurdie and Holmes 2013; Oksanen et al. 2013; Leff 2017). For alpha diversity analysis, richness and Shannon diversity indices were calculated and compared with generalized linear models (GLMs) using microsite as a predictor variable. For beta diversity analysis, Bray–Curtis distances were calculated and ordinated using constrained analysis of principal coordinates (CAPs) with microsite as the constraining variable.

Permutational multivariate analysis of variance (PERMANOVA) models and pairwise comparisons with ‘pairwiseAdonis’ were used to investigate differences in microbial community structure corresponding with microsite (Arbizu 2020). To determine which taxa were differentially abundant between microsites, we used ANCOM‐BC with microsite as the predictor, prevalence cutoff of 0.10, and alpha of 0.05 (Lin and Peddada 2020). Shared taxa were determined using the core_members() function in the ‘microbiome’ package with a detection threshold of 0.001 and prevalence threshold of 0.10 (Lahti and Shetty 2019).

Environmental variables, soil edaphic properties, plant available nutrients, microbial biomass, and MSIR rates were all analyzed in GLMs and analyses of variance (ANOVAs) using microsite as a predictor variable. Spearman rank correlation coefficients were calculated for the dominant microbial taxa, soil properties, and ANPP. All analyses were performed using R version 4.3.0 (R Core Team 2017).

## 3 Results

### 3.1 Aboveground Productivity

Aboveground productivity was highest in E_edge_ (649.03 ± 32.07 g m^−2^), although not significantly higher than Control ANPP. In contrast, aboveground productivity was significantly reduced Beneath panels relative to the Control (378.2 ± 17.56 and 580.20 ± 52.90 g m^−2^, respectively) (*F* = 11.5194, *p* < 0.001, Figure 1b; Tables S1 and S2).

### 3.2 Soil Physiochemistry

Soils at the site of the solar array were classified as clay loam with 39.1% clay, 20.3% silt, and 40.6% sand. Soil pH ranged from 6.97 to 7.82 across microsites and was slightly lower in E_edge_ relative to Control (7.27 ± 0.069 vs. 7.74 ± 0.1196, respectively) (*F* = 3.9613, *p* = 0.018, Tables S1 and S2). Soil organic matter ranged from 0.98% to 1.35% and was significantly higher on the E_edge_ relative to Between (1.22% ± 0.03% vs. 1.03% ± 0.04%, respectively) (*F* = 3.4208, *p* = 0.030, Tables S1 and S2) but did not differ between other microsites. Soil moisture differed between microsites following a pattern previously observed at the same site (*p* < 0.001; Tables S1 and S2). Bulk density, total C, total N, and EC did not differ across microsite treatments or control (Tables S1 and S2).

Growing season soil nutrient availability indicated distinct microsite dynamics across the early (May and June) and late (July and August) growing season. Early growing season nutrient availability was assessed within the microsites directly affected by PV panels (i.e., Beneath, E_edge_, W_edge_, Table S3). Of the macronutrients, only P (*F* = 16.052, *p* < 0.001) and K (*F* = 86.443, *p* < 0.001) differed by microsite (Tables S3 and S4). Of the micronutrients, Ca, Mg, Fe, Cu, Zn, S, Al, and Cd all significantly differed by microsite (Tables S3 and S4). Late growing season nutrient availability was assessed across all microsites (including between panels) and the control plots and showed even fewer differences by microsite; of the macronutrients, only K differed across microsites (Tables S3 and S5). Of the micronutrients, only Pb and Cd differed across microsites. Overall, soil nutrient availability generally decreased across PV microsites throughout the growing season (Table S3).

### 3.3 Soil Biotic Properties

#### 3.3.1 Microbial Biomass

**FIGURE 2:**
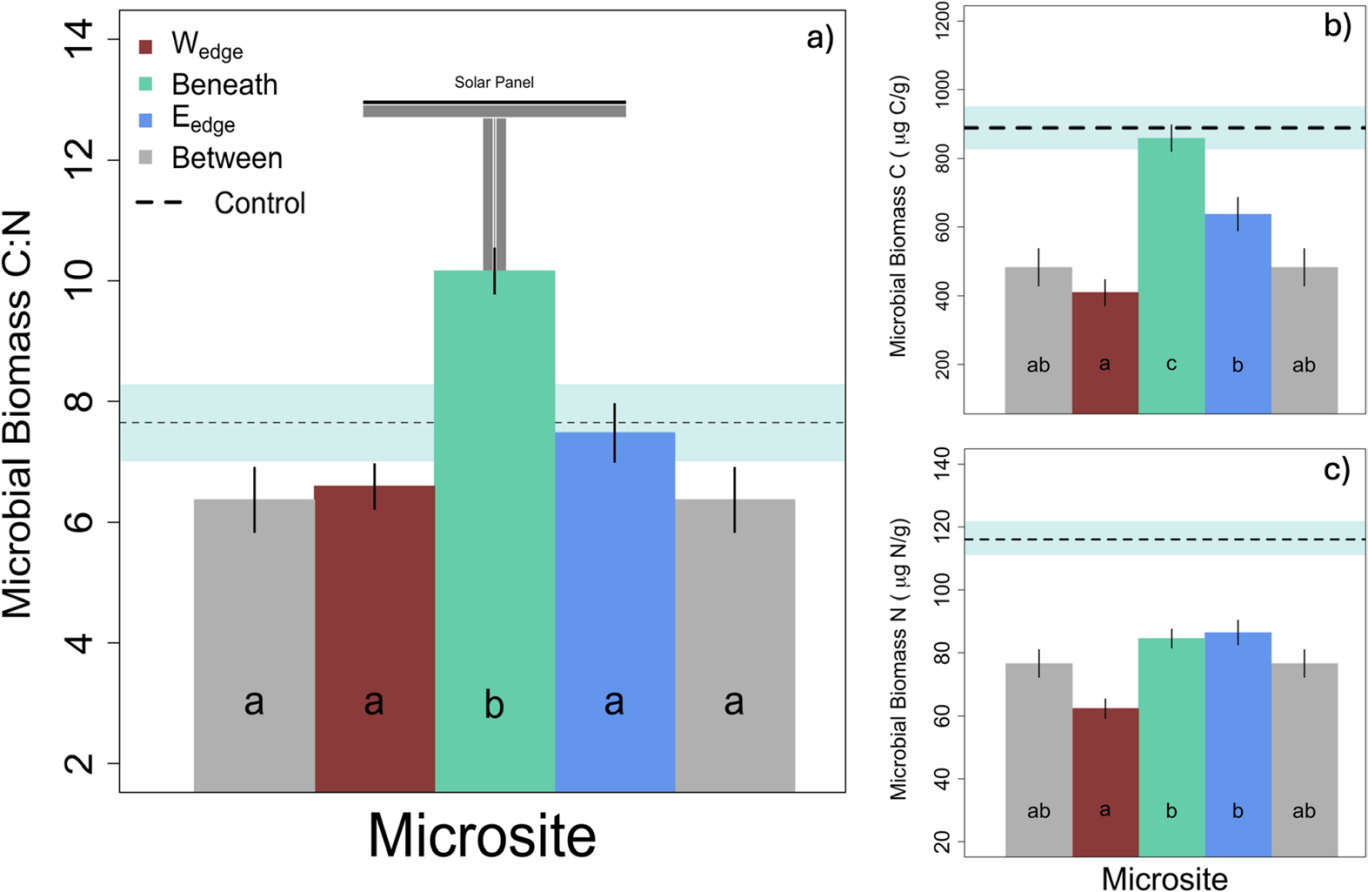
Microbial biomass across microsites. (a) Microbial biomass carbon to nitrogen molar ratios. (b) Microbial biomass C measured in μg g^−1^ dry soil. (c) Microbial biomass N measured in μg g^−1^ dry soil. Lines on vertical bars represent standard error from the mean. Different letters within the bars indicate values that are statistically different (*p* < 0.05, Table S1). The control sample means are represented by a dashed horizontal line with a light blue band representing standard error from the mean.

Microbial biomass carbon (MBC) was lowest in W_edge_ and Between microsites (409 ± 39.3 and 483 ± 55.6 μg C g^−1^ soil, respectively, Figure 2), while highest in Control and Beneath microsites (889 ± 64.3 and 858 ± 39.3 μg C g^−1^ soil, respectively, Figure 2) (*F* = 22.088, *p* < 0.001, Tables S1 and S2). Microbial biomass nitrogen (MBN) was lowest in W_edge_ and Between (62.3 ± 3.18 and 76.6 ± 4.50 μg C g^−1^ soil, respectively, Figure 2), and highest in Control and E_edge_ (116.6 ± 5.20 and 86.5 ± 4.03 μg C g^−1^ soil, respectively, Figure 2) (*F* = 21.343, *p* < 0.001, Tables S1 and S2), whereas microbial biomass C:N ratios were lowest in Between and W_edge_ (6.37 ± 0.31 and 6.59 ± 0.21 μg C g^−1^ soil, respectively, Figure 2) and highest in Beneath and Control (10.16 ± 0.57 and 7.65 ± 0.56 μg C g^−1^ soil, respectively, Figure 2; Table S2).

#### 3.3.2 Multiple Substrate Induced Respiration (MSIR, MicroResp)

**FIGURE 3:**
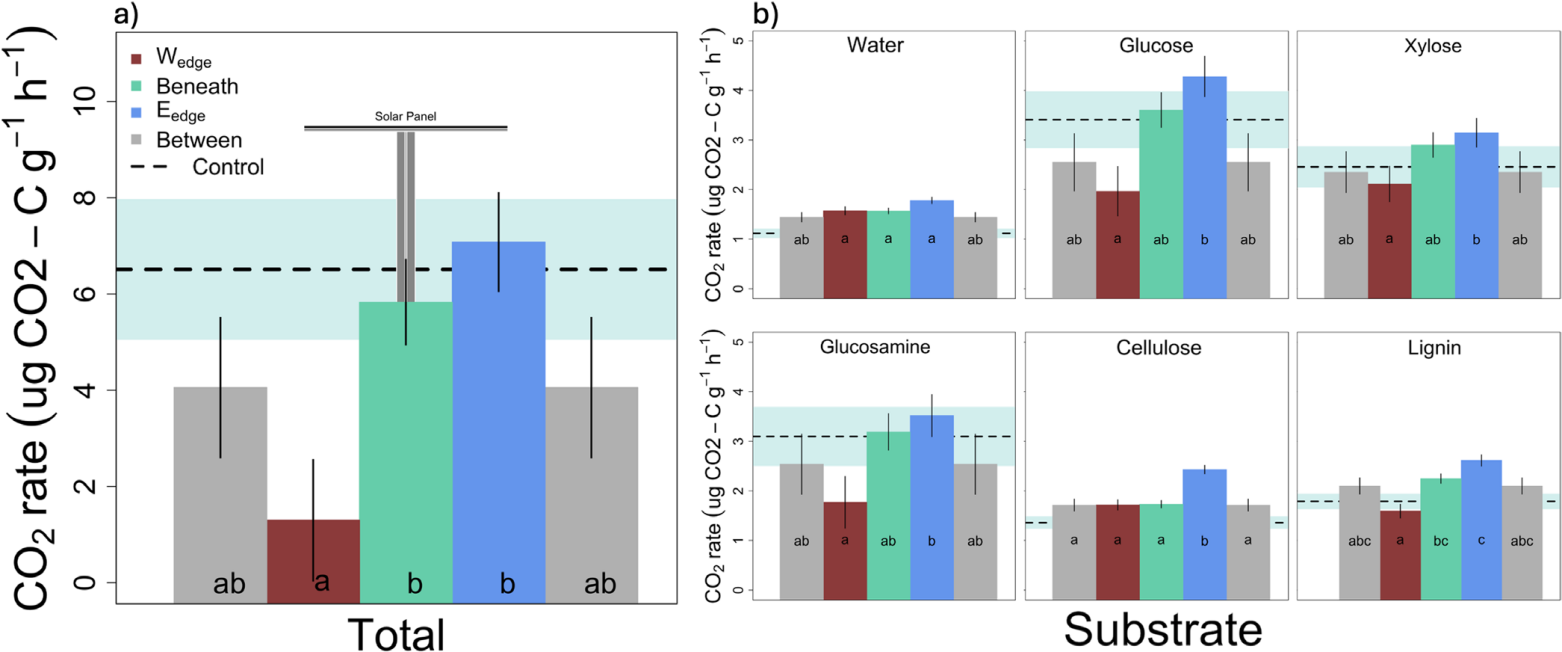
Mean response of soil respiration to six substrates across microsites. (a) Cumulative mean respiration responses to all six substrates (i.e., total respiration) after subtracting water controls (i.e., basal respiration). (b) Soil respiration rates under substrate addition of increasingly recalcitrant organic substrates. Individual bars follow spatial distribution of microsite sampling scheme (Figure 1). Lines on vertical bars represent standard error from the mean. Different letters within the bars indicate values that are statistically different (*p* < 0.05). The control sample means are represented by a dashed horizontal line with a light blue band representing standard error from the mean.

Similar to MBC and MBN, overall MSIR rates were significantly lower in W_edge_ (1.30 ± 0.27 μg g^−1^ h^−1^ CO_2_‐C) relative to all other microsites, with highest rates observed in E_edge_ and Control (7.08 ± 1.22 and 6.51 ± 1.53 μg g^−1^ h^−1^ CO_2_‐C respectively) (*F* = 3.6452, *p* = 0.012, Tables S1 and S6). Basal (water) respiration displayed alternative trends, with rates significantly lowest in Control (1.12 ± 0.09 μg g^−1^ h^−1^ CO_2_‐C) relative to all other microsites and highest rates observed in the E_edge_ microsite (1.78 ± 0.07 μg g^−1^ h^−1^ CO_2_‐C) (*F* = 7.1546, *p* < 0.001). The E_edge_ microsites consistently displayed the highest respiration rates across every substrate, while the lowest rates were substrate dependent (Figure 3; Table S6).

### 3.4 Soil Microbial Community Composition

#### 3.4.1 Alpha Diversity

**FIGURE 4:**
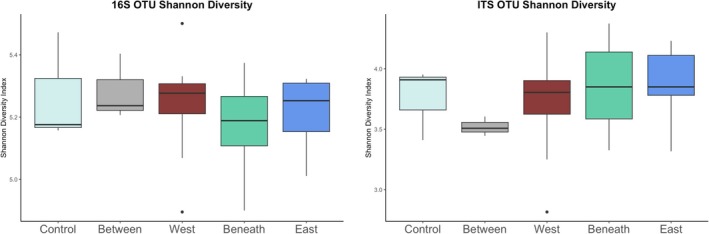
(Left) Shannon diversity of bacteria, archaea, and (right) fungi. Boxes represent the median and interquartile range. Whiskers represent the maximum and minimum values. There are no significant differences for either amplicon.

There were no significant differences in bacterial and archaeal Shannon diversity across microsites (Figure 4; Table S5). However, Between and Control microsites had the highest Shannon diversity (5.29 ± 0.05 and 5.27 ± 0.11, respectively), and Beneath and E_edge_ microsites had the lowest Shannon diversity (5.17 ± 0.04 and 5.22 ± 0.03, respectively).

There were no significant differences in fungal Shannon diversity across microsites, although trends were more pronounced relative to bacteria/archaea and differed substantially (Figure 4; Table S5). The E_edge_ and Beneath microsites had the highest Shannon diversity (3.86 ± 0.09 and 3.85 ± 0.14, respectively), and Between and W_edge_ had the lowest Shannon diversity (3.52 ± 0.05 and 3.70 ± 0.13, respectively).

#### 3.4.2 Beta Diversity

**FIGURE 5:**
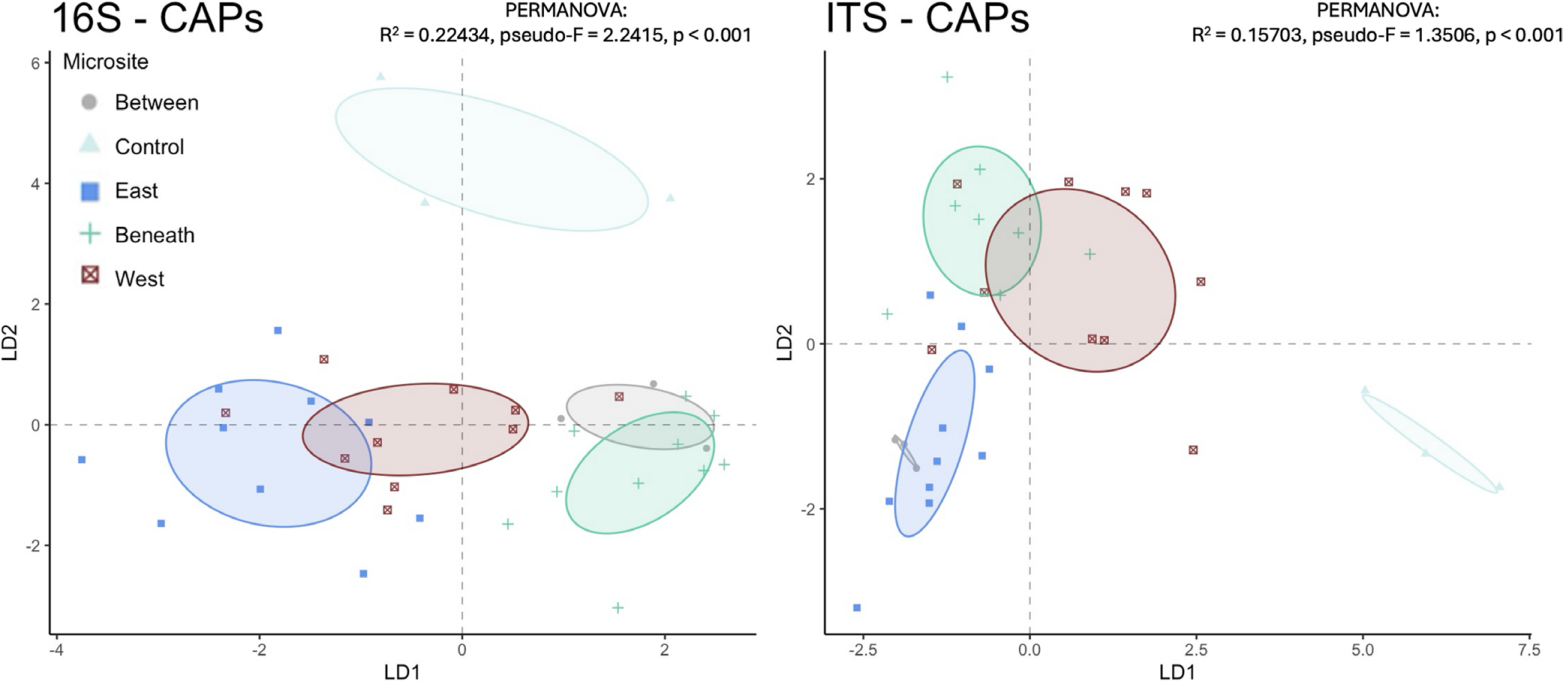
Ordinations for canonical analysis of principal coordinates (CAP) illustrating the impact of microsite heterogeneity on (left) soil bacterial, archaeal, and (right) fungal communities. CAP analyses based on Bray–Curtis dissimilarities. Permutational multivariate analysis of variance (PERMANOVA) model results displayed in upper corner of each panel. CAP was conducted following detection of the significant microsite effects via PERMANOVA (*p* < 0.05).

Prokaryotic (bacterial and archaeal) communities differed by microsite (pseudo‐*F* = 2.2415, *p* < 0.001, Figure 5; Table S5). Differences were most pronounced between the Control and W_edge_ (*p* = 0.06), Beneath (*p* = 0.05), and E_edge_ (*p* = 0.03) microsites according to pairwise analyses. Analysis of the E_edge_ and Between communities also suggested differences in composition (*p* = 0.09).

Fungal communities also differed by microsite (pseudo‐*F* = 1.3506, *p* < 0.001, Figure 5; Table S5). Differences were most pronounced between the Control and W_edge_ (*p* = 0.025), Beneath (*p* = 0.026), and E_edge_ (p = 0.025) microsites according to pairwise analyses. In brief, Control prokaryotic and fungal communities differed substantially from those of microsites that are most directly influenced by panels (i.e., E_edge_, Beneath, W_edge_).

#### 3.4.3 Taxonomic Differences

**FIGURE 6:**
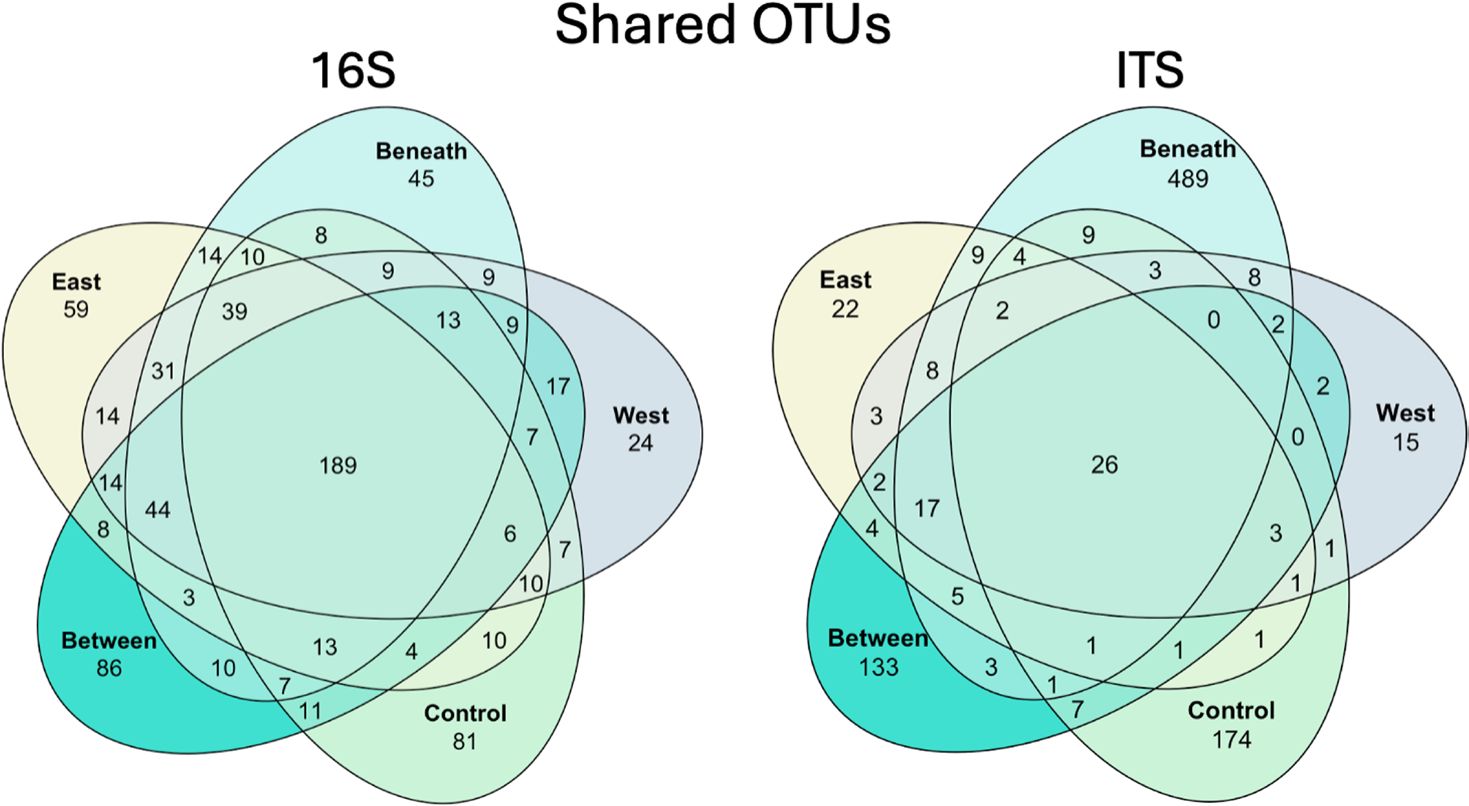
Number of shared and unique OTUs between microsites. Values associated only with a single microsite indicate OTUs that are unique to that microsite. Values in overlapping circles indicate taxa that are shared between microsites.

**FIGURE 7:**
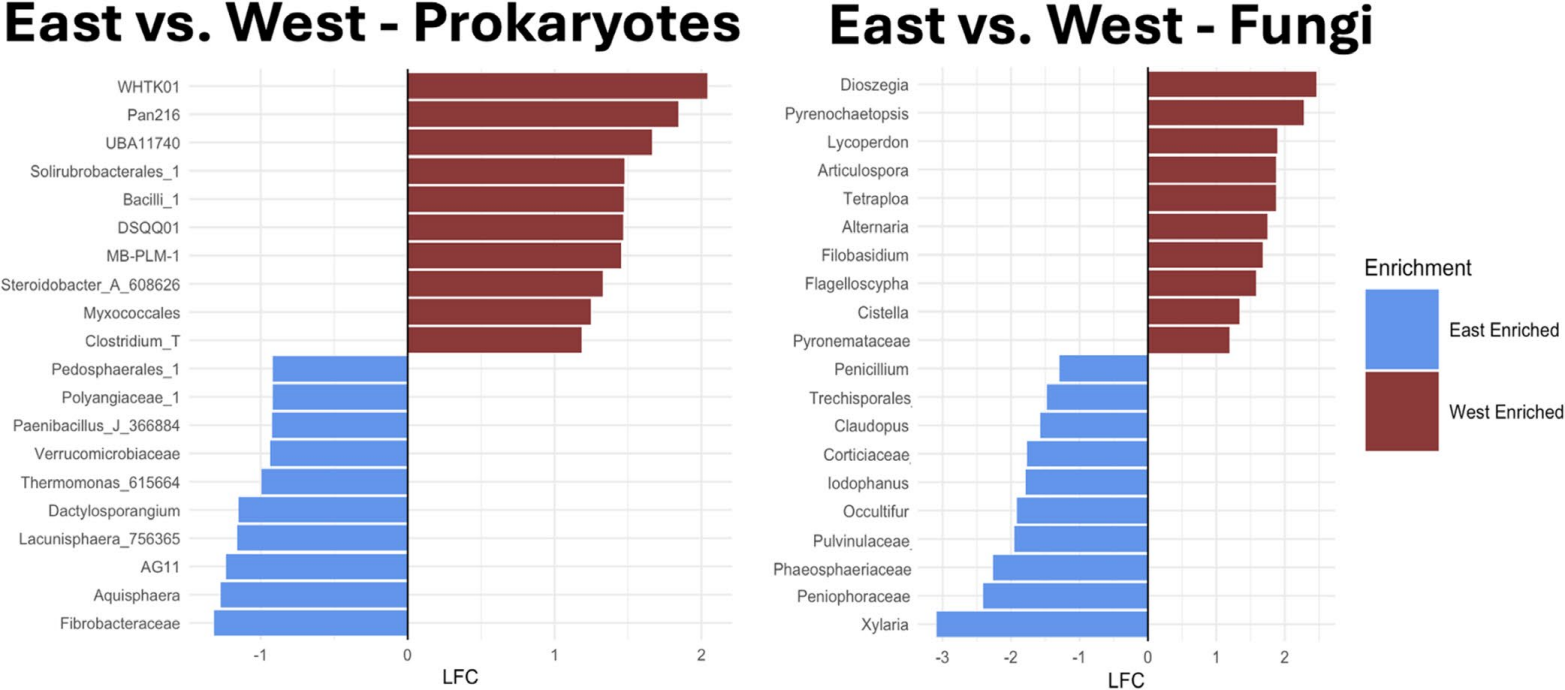
Differentially abundant taxa in the east and west edge microsites. (Left) Bacterial and archaeal taxa that are enriched in the E_edge_ (blue) and W_edge_ (red) at the genus level or finest available taxonomic resolution. (Right) Fungal taxa that are enriched in the E_edge_ (blue) and W_edge_ (red) at the genus level or finest available taxonomic resolution. Differential abundance analysis was conducted with the ANCOM‐BC R package, and logfold changes are visualized along the *x*‐axis.

The most abundant bacterial and archaeal classes across all samples included Nitrososphaeria (20.7%), Vicinamibacteria (14.9%), Verrucomicrobiae (10.5%), Planctomycetia (8.5%), Alphaproteobacteria (6.4%), Bacteroidia (5.9%), and Actinomycetia (5.2%) by relative abundance (see Figure S2 for phylum‐level breakdown). The top 10% of dominant OTUs accounted for 66.1% of total reads, with the ammonia‐oxidizing archaea *Nitrosocosmicus hydrocola* accounting for 12.3% of total reads alone. One hundred and eighty‐nine OTUs were shared across all microsites; 81 were unique to Control, 86 unique to Between, 59 unique to E_edge_, 45 unique to Beneath, and 24 unique to W_edge_ (Figure 6). Among the dominant bacterial and archaeal classes, Verrucomicrobiae differed by microsite (*p* = 0.002, relative abundances in Between = 4.3%, Control = 8.3%, E_edge_ = 8.3%, Beneath = 5.4%, W_edge_ = 7.4%), along with Planctomycetia (*p* = 0.009, relative abundances in Between = 9.2%, Control = 8.8%, E_edge_ = 9.7%, Beneath = 7.5%, W_edge_ = 9.1%). The most enriched bacterial and archaeal taxa in the E_edge_ were Fibrobacteraceae, Enterobacteraceae, and Pedosphaerales relative to the W_edge_. Conversely, the most enriched bacterial and archaeal taxa in the W_edge_ were family WHTK01, family Pan216, and Solirubrobacterales relative to the E_edge_ (Figure 7). These explicit comparisons were made based on our hypothesized and observed differences in decomposition rates.

The most abundant fungal classes across all samples included Mortierellomycetes (18.3%), Pezizomycetes (13.5%), Agaricomycetes (13.5%), Tremellomycetes (10.1%), Leotiomycetes (9.9%), and Glomeromycetes (5.6%) by relative abundance (see Figure S3 for phylum‐level breakdown). The top 10% of dominant OTUs accounted for 76.8% of total reads, with the most dominant OTU, *Pulvinula* sp., accounting for 5.1% of total reads alone. 26 OTUs were shared across all microsites; 176 were unique to Control, 133 unique to Between, 22 unique to E_edge_, 489 unique to Beneath, and 14 unique to W_edge_ (Figure 6; Figure S4). Among the dominant fungal classes, Agaricomycetes differed by microsite (*p* = 0.002, relative abundances in Between = 12.2%, Control = 12.6%, E_edge_ = 13.9%, Beneath = 3.4%, W_edge_ = 6.8%), along with Dothideomycetes (*p* = 0.08, relative abundances in Between = 2.9%, Control = 7.8%, E_edge_ = 15.4%, Beneath = 16.9%, W_edge_ = 16.9%). The most enriched fungal taxa in the E_edge_ were *Xylaria*, Peniophoraceae, and Phaeosphaeriaceae relative to the W_edge_. Conversely, the most enriched fungal taxa in the W_edge_ were Dioszegia, Pyrenochaetopsis, and Lycoperdon relative to the E_edge_ (Figure 7).

## 4 Discussion

We assessed how a single‐axis tracking PV array altered soil microbial communities and physiochemical properties in a semi‐arid grassland. We found that PV‐induced heterogeneity promoted unique soil microbial community structure across PV microsites and relative to non‐PV controls, with overall differences largely dictated by shifts in the relative abundance of fungal taxa. We also found that, within the array, microbial biomass was highest beneath panels, where the most unique fungal taxa were located, while microbial activity (measured as substrate decomposition) was highest along the eastern panel edge (E_edge_), where organic matter (OM) and aboveground net primary production (ANPP) were highest. Subtle differences in soil physiochemical profiles contributed to patterns of soil microbial community structure and activity, but the lack of major profile shifts indicates the importance of PV array construction practices that avoid disturbance and conserve existing biological function.

Spatial differences in plant productivity and soil moisture within the PV array were consistent with previous findings in a semi‐arid climate, where E_edge_ plants were most productive, while W_edge_ soils remained the wettest throughout the growing season (Sturchio et al. 2022; Kannenberg et al. 2023; Sturchio, Kannenberg, Pinkowitz, and Knapp 2024). As expected, beneath panels remained driest and had the lowest ANPP. Because of these trends, we anticipated that soil microbial biomass would differ most substantially across W_edge_ and beneath panel microsites. Our hypothesis was supported by the magnitude of differences; however, the directionality of this relationship, where microbial biomass (C and N) was greatest beneath panels, was unexpected (Figure 2). The exceptionally wet 2023 growing season (536 mm, +46% greater than long term average), where SM did not drop below 25% beneath panels, may have prevented water limitation and thus facilitated microbial proliferation. Another proposed mechanism for increased microbial biomass accumulation beneath panels is greater belowground C allocation under persistent shading (Bahn et al. 2013; Moscatelli et al. 2022), although others have found the opposite response to increased shading (Möhl et al. 2020). Surprisingly, microbial biomass was higher in control plots than in any PV microsite (Figure 2). Given the control plots are unimpeded by PV panels, they undergo the longest daily photoperiods, potentially increasing the production of plant photosynthetic products and altering root exudate composition and concentration (Vives‐Peris et al. 2020). Unique exudate composition or increased concentrations could enhance microbial biomass production in control plots relative to PV microsites. The lower microbial biomass within a PV array is corroborated by Lambert et al. (2021) who found lower microbial biomass in a PV array relative to reference pinewood and shrubland, though the PV array had been graded during construction.

Microbial activity did not consistently parallel patterns of microbial biomass; rather, it was more closely related to changes in soil physiochemical properties. The E_edge_ harbored the highest microbial activity (Figure 3), likely influenced by the relatively high OM and ANPP and neutral pH. These findings are consistent with previous assessments of microbial activity in natural ecosystems indicating pH and organic C as primary determinants of activity (Creamer et al. 2016; Fierer and Jackson 2006; Nielsen et al. 2010). Although OM is a proxy of organic C, relatively high OM, ANPP, moderate SM, and neutral pH at the E_edge_ generated an environment ideal for turnover of organic compounds within the array. The rapid degradation of both labile (e.g., glucose) and recalcitrant (e.g., lignin) compounds indicates that E_edge_ microbial communities contain a wide array of metabolic pathways (Neely et al. 1991). Interestingly, W_edge_ had the lowest microbial activity, likely due to extended periods of > 40% SM that may have caused waterlogging and oxygen limitation, which may have reduced microbial biomass, limited microbial activity, and altered community structure in the semi‐arid system that is accustomed to drier, aerobic soils (Mentzer et al. 2006; Blagodatsky and Smith 2012; Banerjee et al. 2016; Liu et al. 2023).

In natural systems, differences in microbial activity and metabolic diversity have been attributed to shifts in microbial community composition (Hernández and Hobbie 2010; Xun et al. 2015). In further support of our hypothesis, microbial community composition differed across PV microsites, with the most notable differences between PV and non‐PV control communities. Although prokaryotic communities were predominantly composed of the globally ubiquitous soil phyla, Acidobacteria, Thermoproteota, Actinobacteria, Planctomycetes, and Proteobacteria (Figures S2 and S5, Delgado‐Baquerizo et al. 2018), shifts in the relative abundance of the phyla Verrucomicrobia and Planctomycetia drove differentiation between communities. These findings contrast with those of a semi‐arid grassland in China, where Actinobacteria and Proteobacteria were the primary drivers of compositional shifts (Bai et al. 2022). Furthermore, prokaryotic communities were dominated by the ammonia‐oxidizing archaea, *Nitrosocosmicus hydrocola*, which is likely attributable to the history of N fertilization at the site (Huang et al. 2021; Han et al. 2024). Fungal communities were also dominated by ubiquitous phyla, Ascomycota, Basidiomycota, and Mortierellomycota (Tedersoo et al. 2014, Figure S3), but a class‐level analysis suggested that shifts in Agaricomycetes and Dothideomycetes were responsible for driving overall fungal community differences. Although pairwise comparisons did not indicate significant differences in fungal community structure beneath panels and other microsites, the immense number of unique OTUs beneath panels (Figure 6) suggests that this microsite may promote fungal diversity, which could be driven by the hypothesized reallocation of plant photosynthates (Bahn et al. 2013; Moscatelli et al. 2022), differences in soil moisture and nutrient content (Kaisermann et al. 2015), or unique aboveground‐belowground interactions not present in other PV microsites. Interestingly, these findings contrast with Liu et al. (2023), where control fungal communities show no clear differentiation between those within an arid PV array.

Prior studies assessing soil responses to PV arrays tended to focus on soil physiochemical properties that are likely to be negatively impacted by the extreme disturbance associated with PV installation (Lambert et al. 2021; Choi et al. 2024). Without major intervention, these properties, such as total C, are unlikely to return to pre‐disturbance conditions for years to decades (Jackson et al. 2017). While soil physiochemical properties may not differ drastically across PV microsites if minimally invasive construction practices are leveraged, soil biological processes, major controls over ecosystem C‐cycling, nutrient cycling, and overall functionality, can still differ across relatively small spatial scales (Liu et al. 2023; Leroy et al. 2025). For example, the fungal genus, *Xylaria*, was enriched along the E_edge_ (relative to W_edge_), promoting higher rates of recalcitrant substrate decomposition (Figures 5 and 7, Osono et al. 2011). Our findings corroborate recent studies of soil microbial activity in PV arrays (Moscatelli et al. 2022; Liu et al. 2023; Lambert et al. 2024), suggesting that, even across different ecosystems, PV arrays substantially alter belowground functionality. Collectively, these findings provide evidence of PV‐induced environmental heterogeneity contributing to soil microbial niche differentiation, which may be increasingly evident under more extreme climatic conditions (Sturchio and Knapp 2023).

The recent surge of studies assessing ecosystem impacts of PV arrays has been invaluable for informing the sustainable development of renewable energy infrastructure, yet the complexity of ecological interactions necessitates scalable approaches that can integrate data from disparate ecosystem types (e.g., simulation models, Paschalis et al. 2025). To understand how this form of land‐use change may impact carbon and nutrient cycling, such approaches should leverage case studies that capture the impacts of various PV array designs, construction practices, and management types globally (Bacon et al. 2024). The data presented here suggest that landscape‐scale models and future case studies should consider the unique processes underway at different PV microsites. While these data highlight the importance of taking a minimally invasive approach (e.g., no land grading) to PV array construction to preserve ecosystem functionality and limit the need for restoration practices, we further suggest that restoration approaches within PV arrays take advantage of the contrasting conditions present across PV microsites, which may facilitate the establishment of different plant communities, enhanced soil biodiversity, and potentially soil C sequestration (Lambert et al. 2024; Krasner et al. 2025; Sturchio and Knapp 2025).

## 5 Conclusion

The rapid pace and scale of PV array installations has generated concern regarding the ecosystem consequences of such a large‐scale land use change (Cook 2011; Hernandez et al. 2014). Here, we show that, following a minimally invasive array construction, PV‐induced changes in microenvironmental conditions can substantially alter soil microbial community structure and function and, to a lesser degree, soil physiochemical properties, across relatively small (m^2^) spatial scales. Although microsite differences in most soil physiochemical properties were modest, microbial community structure and function were strongly influenced by the heterogeneity induced by PV panels. PV‐driven shifts in soil functionality have the potential to drive large‐scale changes in ecosystem functionality, from altered plant nutrient availability to C fluxes. Considering the diversity of PV construction practices and designs, it is imperative that we conduct further investigations of belowground responses to better understand how global C and nutrient cycles will respond to this form of land use change. We suggest future studies pair analyses of soil properties with soil microbial structure and function to provide clearer insight into short and long‐term processes that cannot be gauged by any single measure. These approaches should leverage metagenomics, metatranscriptomics, and other‐omics technology where possible to generate a more holistic picture of soil food web contributions to belowground functionality. We further recommend that sampling be conducted across discrete PV microsites that are likely to provide distinct ecosystem functions (e.g., W_edge_, E_edge_, Beneath, Between), rather than a single PV microsite (i.e., beneath panels). PV arrays also provide abundant potential to investigate the environmental controls over plant–soil interactions. Given this growing form of land use is primed to become a primary source of energy production globally (Nijsse et al. 2023), accelerating belowground research and emphasizing more sustainable construction practices must be a priority.

## Author Contributions

**J. Alexander Siggers:** conceptualization, data curation, formal analysis, funding acquisition, investigation, methodology, project administration, resources, software, validation, visualization, writing – original draft, writing – review and editing. **Matthew A. Sturchio:** conceptualization, data curation, formal analysis, funding acquisition, investigation, methodology, project administration, resources, supervision, visualization, writing – original draft, writing – review and editing. **Lillian Gordon:** data curation, investigation, methodology, writing – review and editing. **Shelby Mead:** investigation, methodology, writing – review and editing. **Melinda D. Smith:** supervision, writing – review and editing. **Alan K. Knapp:** funding acquisition, project administration, resources, supervision, writing – original draft, writing – review and editing.

## Conflicts of Interest

The authors declare no conflicts of interest.

## Supporting information


Data S1.

## Data Availability

The data and code that support the findings of this study are openly available in Zenodo at http://doi.org/10.5281/zenodo.15192185. Raw sequences are available at NCBI accession number: PRJNA1280198.
