# Supplementary material for: Environmental Heterogeneity Imposed by Photovoltaic Array Alters Grassland Soil Microbial Communities
Source: Glob Chang Biol. 2025 Jul 23;31(7):e70376. doi: 10.1111/gcb.70376 (PMC12285215; doi:10.1111/gcb.70376)
Supplement: Supplementary file 1 — Data S1. [file GCB-31-e70376-s001.pdf]

<u>**Supplemental Materials**</u>

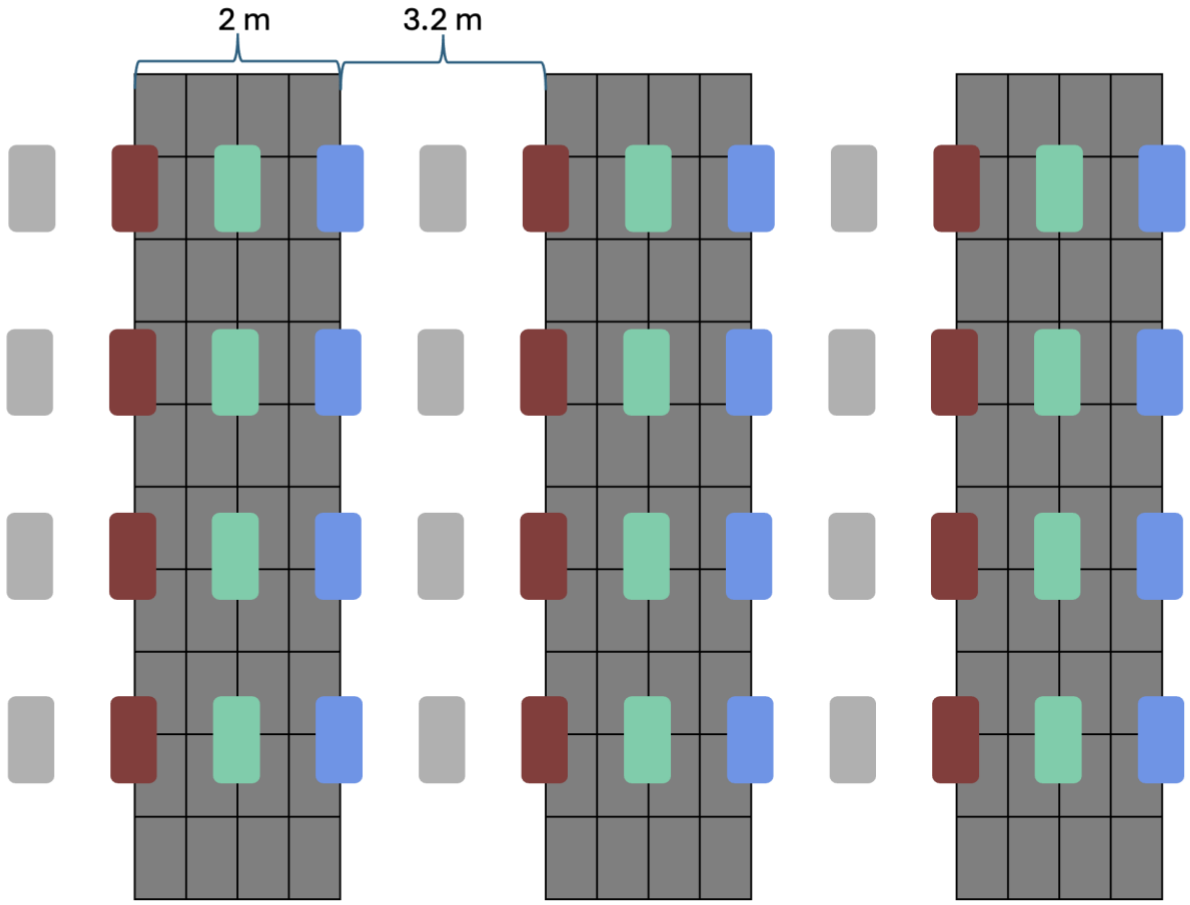

Figure S1: Conceptual figure of sampling scheme. Experimental microsites are color coded to match the primary manuscript. *Between* is grey, W_{edge} is red, *Beneath* is green, and E_{edge} is blue.

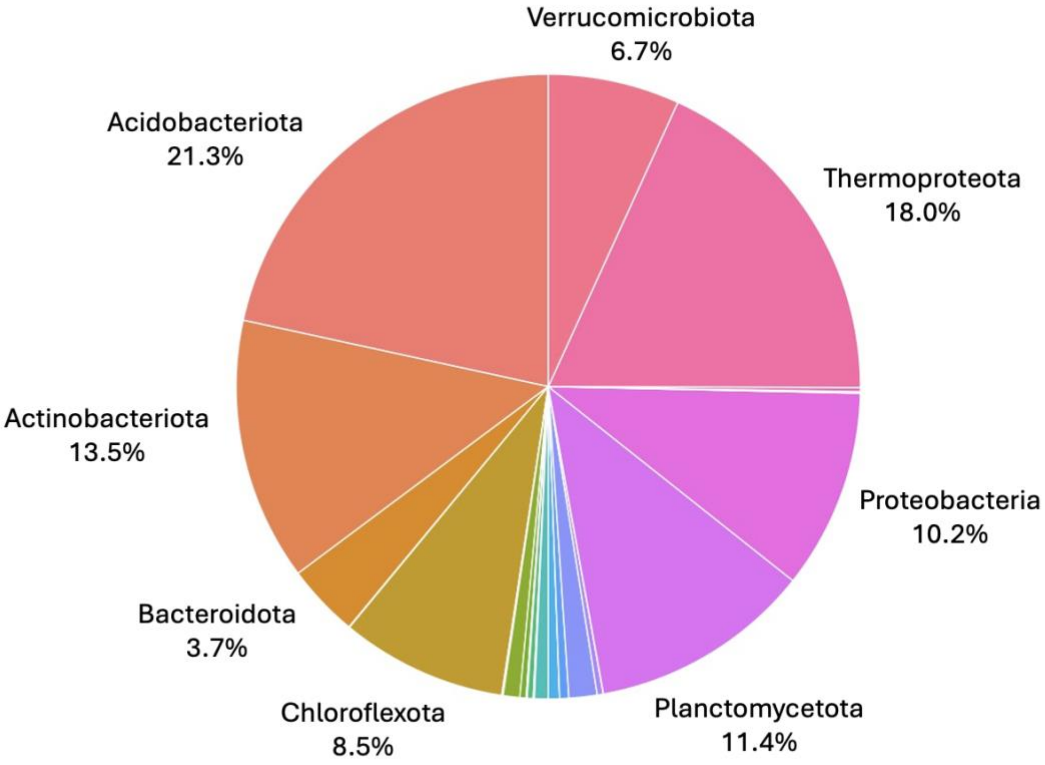

Figure S2: Relative abundance of dominant bacterial/archaeal phyla averaged across all samples.

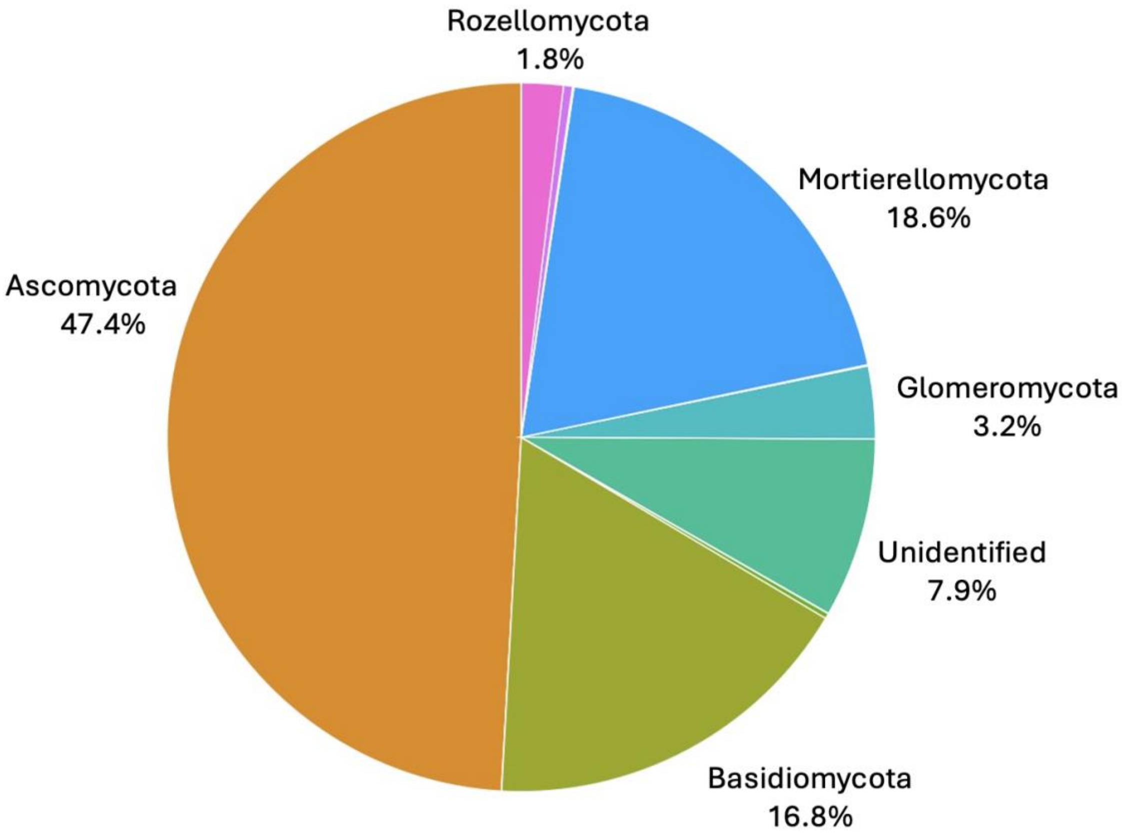

Figure S3: Relative abundance of dominant fungal phyla averaged across all samples.

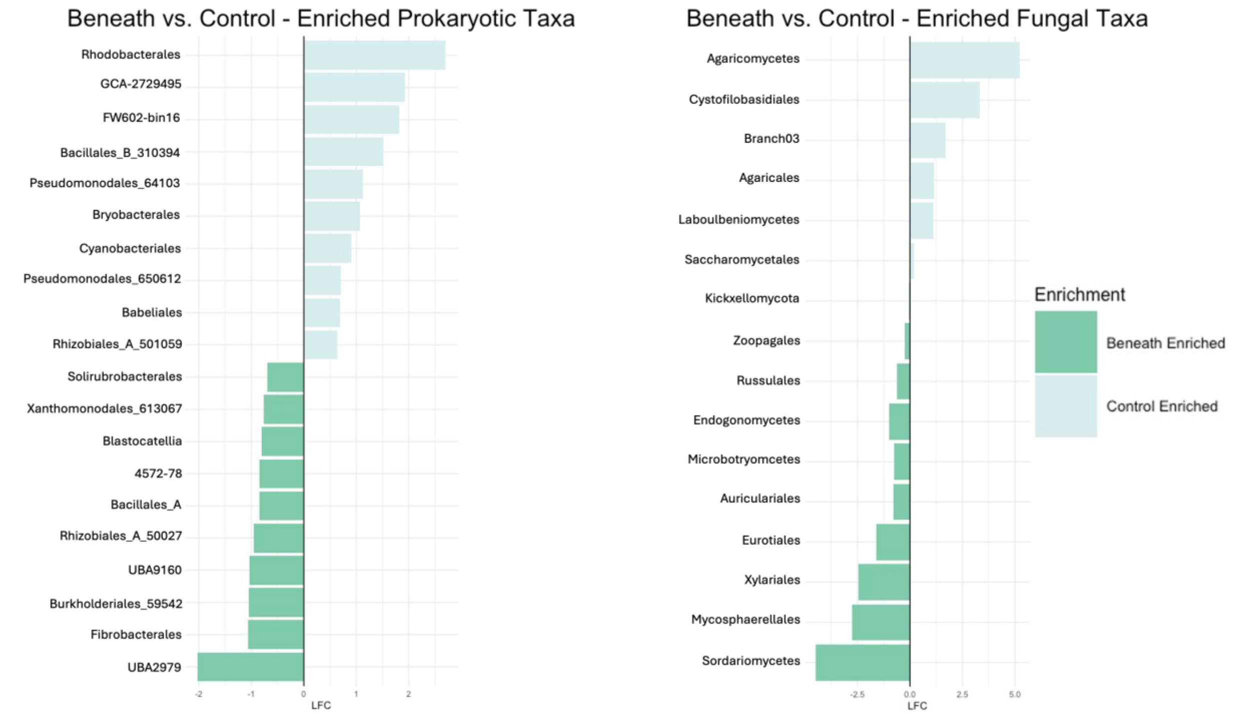

Figure S4: Differentially abundant taxa in the Beneath panel and Control microsites. a): Bacterial and archaeal taxa that are enriched in Beneath (aquamarine) and Control (cyan) at the order level. b): Fungal taxa that are enriched in Beneath (aquamarine) and Control (cyan) at the order level. Differential abundance analysis was conducted with the ANCOM-BC R package, and logfold changes are visualized along the x-axis.

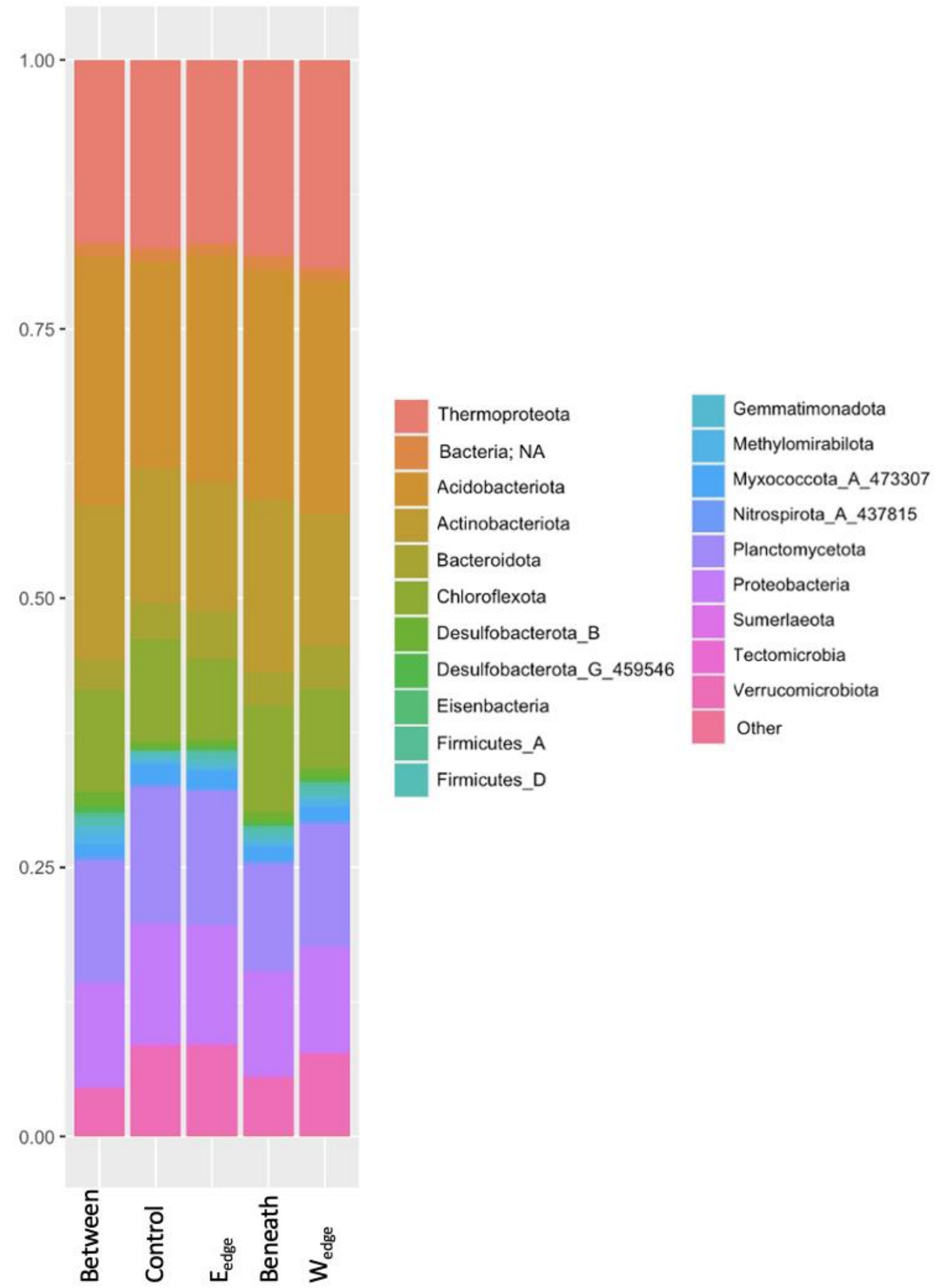

Figure S5: Relative abundance of the 20 most abundant prokaryotic phyla in each microsite.

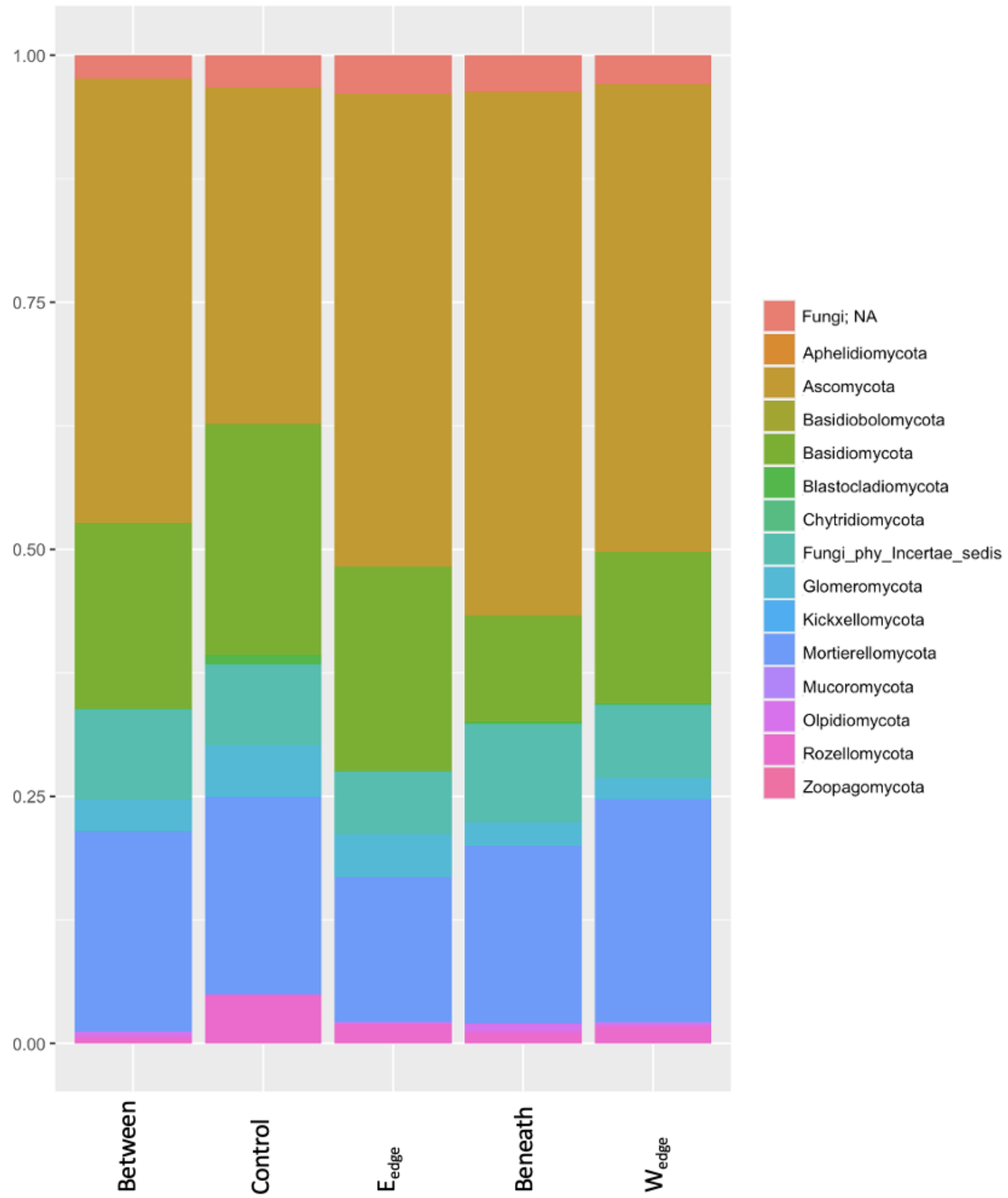

Figure S6: Relative abundance of the 14 most abundant fungal phyla in each microsite.

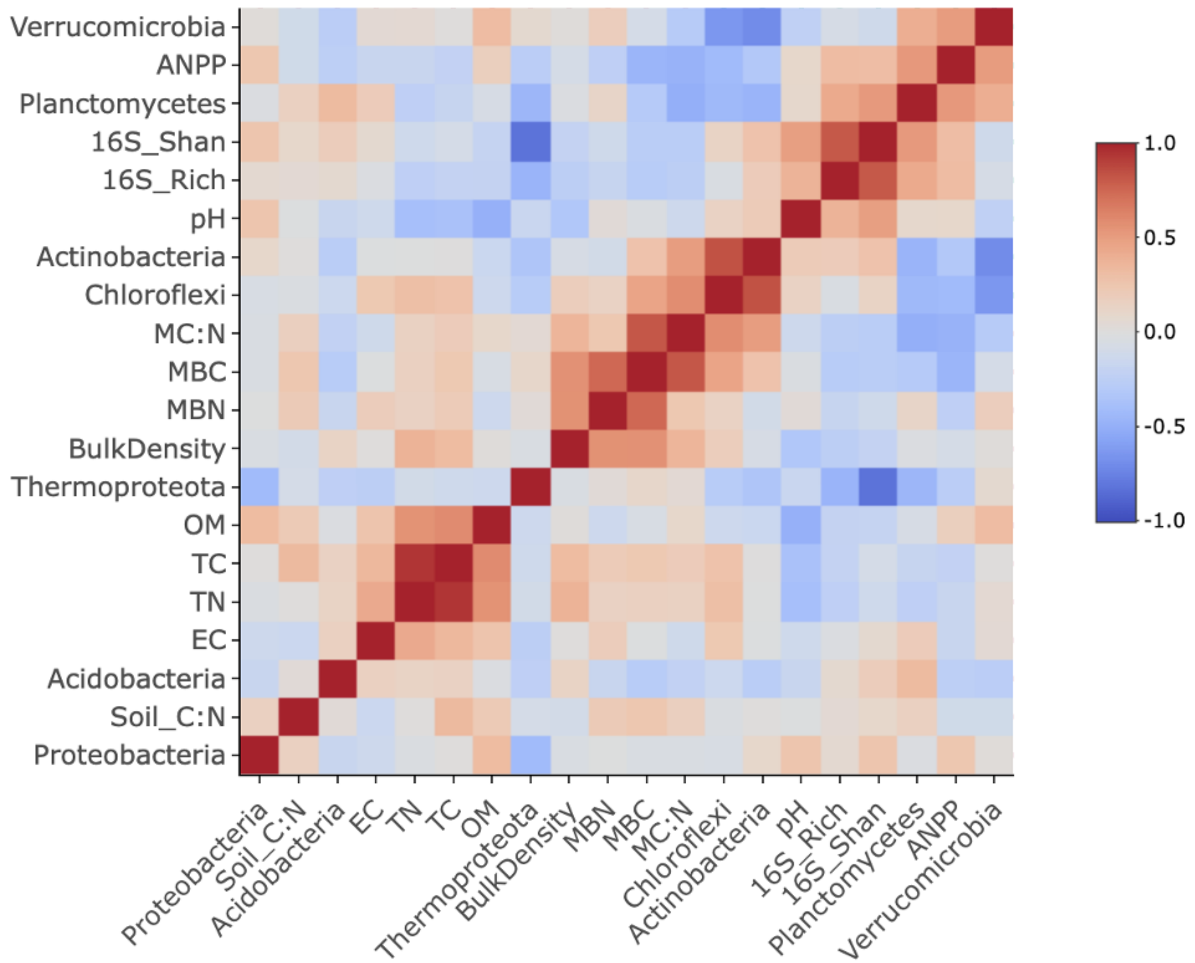

Figure S7: Spearman rank correlation analysis of dominant bacterial and archaeal phyla and soil properties. ANPP = Aboveground Net Primary Productivity, 16S_Shan = Shannon diversity of bacteria & archaea, 16S_Rich = Richness of bacteria & archaea, MC:N = Microbial biomass C:N ratio, MBC = Microbial biomass C, MBN = Microbial biomass N, OM = Organic Matter, TC = Total C, TN = Total N, Soil_C:N = Soil C:N ratio.

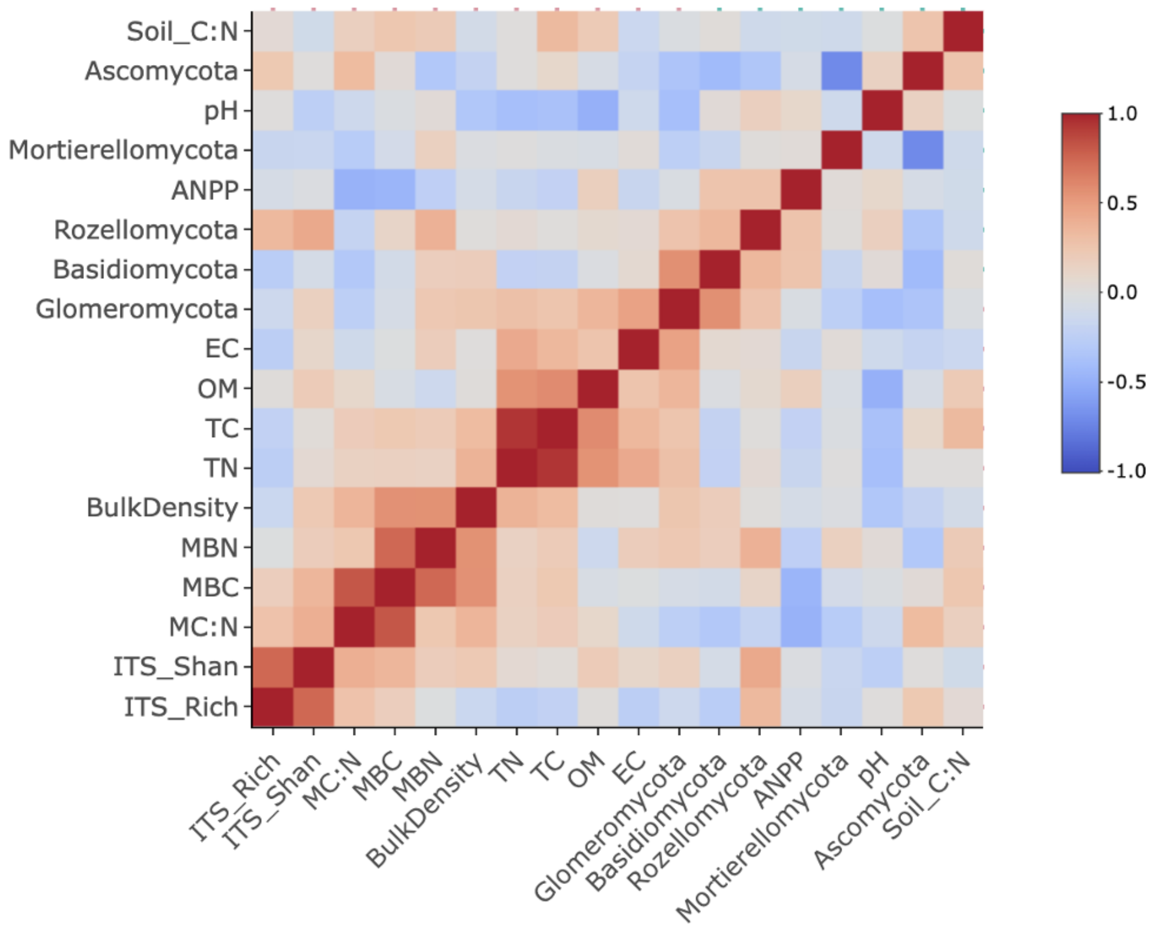

Figure S8: Spearman rank correlation analysis of dominant fungal phyla and soil properties. ANPP = Aboveground Net Primary Productivity, ITS_Shan = Shannon diversity of fungi, ITS_Rich = Richness of fungi, MC:N = Microbial biomass C:N ratio, MBC = Microbial biomass C, MBN = Microbial biomass N, OM = Organic Matter, TC = Total C, TN = Total N, Soil_C:N = Soil C:N ratio.

Response Variable	Microsite	
	df	F
ANPP	4	11.5194***
Organic Matter	4	3.4208*
Electrical Conductivity	4	0.5925
Total C	4	1.489
Total N	4	0.9818
pH	4	3.9613*
Microbial Biomass C	4	22.088***
Microbial Biomass N	4	21.343***
Microbial Biomass C:N	4	13.56***
Water	4	7.1546***
Glucose	4	3.7322*
Cellulose	4	14.384***
Xylose	4	1.6093
Glucosamine	4	1.8988
Lignin	4	8.7431***
Overall	4	3.6452*

Table S1: ANOVA tests of environmental and soil physiochemical properties, microbial biomass, and MicroResp substrate induced respiration rates using microsite as a predictor. *p<0.05; **p<0.01; ***p<0.001.

Response Variable	Microsite				
	Between	E_{edge}	Beneath	W_{edge}	Control
ANPP (g/m^2)	535.71 ± 38.57	649.03 ± 32.07	378.15 ± 17.56	555.19 ± 24.49	580.20 ± 52.90
Average Growing Season Soil Moisture (0-15cm)	36.1%	36.7%	28.3%	41.5%	37.2%
Average Sampling Day Soil Moisture (0-15cm)	30.4%	30.2%	27.3%	45.5%	33.8%
Organic Matter (OM)	1.03% ± 0.04	1.22% ± 0.03	1.17% ± 0.03	1.15% ± 0.03	1.10% ± 0.05
Electrical Conductivity (EC) (dS/m)	0.327 ± 0.04	0.288 ± 0.03	0.303 ± 0.03	0.297 ± 0.03	0.370 ± 0.05
Total C (TC)	2.63% ± 0.17	2.90% ± 0.12	3.02% ± 0.12	2.67% ± 0.12	2.85% ± 0.21
Total N (TN)	2.33% ± 0.01	2.45% ± 0.01	2.55% ± 0.01	2.28% ± 0.01	2.40% ± 0.02
pH	7.51 ± 0.10	7.27 ± 0.07	7.37 ± 0.07	7.54 ± 0.07	7.74 ± 0.12
Bulk Density (g/cm^3)	1.071	1.184	1.149	0.929	1.113
Microbial Biomass C (MBC) (μg C g^{-1})	482.72 ± 15.89	637.37 ± 70.97	858.34 ± 49.85	408.76 ± 21.42	889.18 ± 52.10
Microbial Biomass N (MBN) (μg C g^{-1})	76.60 ± 5.89	86.46 ± 4.26	84.59 ± 2.54	62.29 ± 3.50	116.55 ± 2.49
Microbial Biomass C:N	6.37 ± 0.31	7.48 ± 0.43	10.16 ± 0.57	6.59 ± 0.21	7.65 ± 0.57

Table S2: ANPP and each soil physiochemical property in each microsite with standard error in parentheses. Sampling day was August 2, 2023 for Sampling Day Soil Moisture reference. Bulk density was collected for only two samples in each microsite.

Nutrient	Season	Microsite				
		Between	E_{edge}	Beneath	W_{edge}	Control
NO_3-N (μg/10cm^2/burial length)	Early	NA	35.25 ± 7.61	32.64 ± 12.31	33.87 ± 5.17	297.54
	Late	15.79 ± 4.33	20.88 ± 5.52	41.24 ± 19.01	22.44 ± 2.79	39.58 ± 2.46
	Difference	NA	14.37	-8.60	11.43	257.96
Ca (μg/10cm^2/burial length)	Early	NA	3165.43 ± 63.89	1724.63 ± 247.79	3377.92 ± 67.95	3415.90
	Late	2198.10 ± 369.94	2152.45 ± 219.84	1700.67 ± 414.22	2488.92 ± 158.69	2892.31 ± 40.39
	Difference	NA	1012.98	23.96	889	523.59
Mg (μg/10cm^2/burial length)	Early	NA	301.25 ± 5.63	238.06 ± 22.04	306.11 ± 9.13	236.80
	Late	259.81 ± 30.55	232.63 ± 16.35	203.92 ± 25.37	220.53 ± 10.52	251.73 ± 34.06
	Difference	NA	68.62	34.14	85.58	-14.93
K (μg/10cm^2/burial length)	Early	NA	65.41 ± 4.08	247.42 ± 23.72	60.08 ± 4.11	56.80
	Late	169.70 ± 59.33	128.40 ± 20.98	163.85 ± 39.48	62.63 ± 10.85	252.85 ± 143.35
	Difference	NA	-62.99	83.57	-2.55	-196.05
P (μg/10cm^2/burial length)	Early	NA	53.41 ± 5.42	15.87 ± 3.93	56.16 ± 4.85	2.63
	Late	14.20 ± 4.64	19.38 ± 2.82	12.70 ± 4.22	26.07 ± 4.62	8.82 ± 4.08
	Difference	NA	34.03	3.17	30.09	-6.19

Fe ($\mu g/10cm^2$/burial length)	Early	NA	40.58 ± 5.78	3.34 ± 0.84	47.85 ± 12.07	11.21
	Late	4.83 ± 2.13	5.93 ± 0.94	3.97 ± 0.83	6.68 ± 0.96	9.82 ± 4.55
	Difference	NA	34.65	-0.63	41.17	1.39
Mn ($\mu g/10cm^2$/burial length)	Early	NA	9.99 ± 1.37	1.67 ± 0.37	11.68 ± 1.82	4.01
	Late	1.66 ± 0.66	2.27 ± 0.49	1.04 ± 0.26	2.68 ± 0.77	1.47 ± 0.45
	Difference	NA	7.72	0.63	9	2.54
Cu ($\mu g/10cm^2$/burial length)	Early	NA	3.41 ± 0.18	1.26 ± 0.10	3.75 ± 0.18	2.32
	Late	0.53 ± 0.16	0.45 ± 0.13	0.47 ± 0.12	0.50 ± 0.10	1.09 ± 0.46
	Difference	NA	2.96	0.79	3.25	1.23
Zn ($\mu g/10cm^2$/burial length)	Early	NA	3.41 ± 0.26	0.91 ± 0.21	3.86 ± 0.32	2.54
	Late	0.75 ± 0.18	1.76 ± 0.35	1.15 ± 0.32	2.06 ± 0.52	3.09 ± 1.90
	Difference	NA	1.65	-0.24	1.80	-0.55
B ($\mu g/10cm^2$/burial length)	Early	NA	0.52 ± 0.16	0.24 ± 0.09	0.83 ± 0.35	0.05
	Late	0.24 ± 0.13	0.11 ± 0.03	0.22 ± 0.04	0.14 ± 0.04	0.30 ± 0.16
	Difference	NA	0.41	0.02	0.69	-0.25
S ($\mu g/10cm^2$/burial length)	Early	NA	28.21 ± 2.11	17.42 ± 3.93	34.65 ± 3.91	44.68
	Late	8.74 ± 3.93	13.45 ± 1.46	15.16 ± 6.05	17.73 ± 3.20	31.22 ± 20.35
	Difference	NA	14.76	2.26	16.92	13.46

Pb (μg/10cm^2/burial length)	Early	NA	3.82 ± 0.37	0.22 ± 0.08	4.24 ± 0.43	7.22
	Late	0.51 ± 0.32	0.48 ± 0.13	0.55 ± 0.34	0.87 ± 0.23	4.04 ± 2.90
	Difference	NA	3.34	-0.33	3.37	3.18
Al (μg/10cm^2/burial length)	Early	NA	12.93 ± 1.29	7.83 ± 0.59	13.38 ± 1.80	9.05
	Late	7.92 ± 2.05	7.89 ± 0.55	7.62 ± 0.65	8.78 ± 0.57	9.98 ± 2.17
	Difference	NA	5.04	0.21	4.60	-0.93
Cd (μg/10cm^2/burial length)	Early	NA	3.41 ± 0.18	1.26 ± 0.10	3.75 ± 0.18	2.32
	Late	0.53 ± 0.16	0.45 ± 0.13	0.47 ± 0.12	0.50 ± 0.10	1.09 ± 0.46
	Difference	NA	2.96	0.79	3.25	1.23

Table S3: Early and late growing season nutrient availability in each microsite. Nutrient accumulation was measured with Plant Root Simulator (PRS®) probes (see Methods). Control values were generated from a single set of probes, while the other microsites are expressed as an average of replicates with standard error. The difference between early and late season nutrient availability is expressed as a positive value if nutrient availability was higher in the early growing season.

Response Variable	Microsite	
	df	F
NO3-N	2	0.025
Ca	2	47.624***
Mg	2	9.0189***
K	2	86.443***
P	2	16.052***
Fe	2	5.9551*
Mn	2	1.774
Cu	2	47.848***
Zn	2	25.44***
B	2	1.2593
S	2	5.8078**
Pb	2	1.5008
Al	2	3.5041*
Cd	2	15.796***

Table S4: ANOVA tests of early growing season nutrient availability using microsite as a predictor. *$p<0.05$; **$p<0.01$; ***$p<0.001$.

Response Variable	Microsite	
	df	F
NO_3-N	4	1.0731
Ca	4	1.6584
Mg	4	0.8792
K	4	3.2855*
P	4	2.0455
Fe	4	1.6377
Mn	4	1.0181
Cu	4	1.3705
Zn	4	1.4706
B	4	1.703
S	4	1.518
Pb	4	5.3591**
Al	4	0.811
Cd	4	11.674***

Table S5: ANOVA tests of late growing season nutrient availability using microsite as a predictor. *p<0.05; **p<0.01; ***p<0.001.

Response Variable	Microsite				
	Between	E_{edge}	Beneath	W_{edge}	Control
Water	1.441171 ± 0.068743	1.782780 ± 0.07579	1.569659 ± 0.07559	1.573696 ± 0.05417	1.129289 ± 0.092960
Glucose	2.551994 ± 0.272660	4.281376 ± 0.52157	3.605651 ± 0.42069	1.967145 ± 0.05680	3.415875 ± 0.49467
Cellulose	1.713874 ± 0.08709	2.428955 ± 0.09521	1.734307 ± 0.09821	1.718815 ± 0.02272	1.366398 ± 0.146285
Xylose	2.353103 ± 0.309196	3.147464 ± 0.29985	2.899387 ± 0.33696	2.113969 ± 0.04219	2.466515 ± 0.344060
Glucosamine	2.540205 ± 0.406178	3.519918 (0.37957	3.190252 (0.468531)	1.771337 (0.079882)	3.108473 (0.8114931)
Lignin	2.101027 (0.1474892)	2.617447 (0.102647)	2.246944 (0.118223)	1.594984 (0.05007)	1.799500 (0.2464388)
Overall	4.05327 (1.0267)	7.072516 (1.2212)	5.828246 (1.0272)	1.29777 (0.2704)	6.510316 (1.5336)

Table S6: MicroResp substrate induced respiration rate averages (measured in μg g^{-1} h^{-1} CO_2-C) with standard error in parentheses. Overall was calculated by subtracting water (i.e., basal respiration) from each, then summing.

Response Variable	Microsite	
	df	F
16S Shannon	4	0.5851
16S Bray-Curtis (PERMANOVA)	4	(pseudo-F) 2.2415***
16S Robust Aitchison (PERMANOVA)	4	(pseudo-F) 1.4601***
ITS Shannon	4	0.7470
ITS Bray-Curtis (PERMANOVA)	4	(pseudo-F) 1.3506***
ITS Robust Aitchison (PERMANOVA)	4	(pseudo-F) 1.2339***

Table S7: ANOVA tests of 16S and ITS diversity analyses using microsite as a predictor. *p<0.05; **p<0.01; ***p<0.001.
